# Points of Departure for BPDE-induced cellular senescence and cell death are characterized by altered DNA damage signaling and regulated by unrepaired DNA double-strand breaks

**DOI:** 10.1007/s00204-026-04426-8

**Published:** 2026-05-21

**Authors:** Ariane Schmidt, Anna Schöneis, Jason Sallbach, Alexandra Pöschmann, Rebekka Lippold, Birgit Rasenberger, Thomas G. Hofmann, Maja T. Tomicic, Markus Christmann

**Affiliations:** Department of Toxicology, University Medical Center of the Johannes Gutenberg University of Mainz, Obere Zahlbacher Str. 67, 55131 Mainz, Germany

**Keywords:** Benzo[a]pyrene, DNA damage response (DDR), DNA damage, Cellular senescence, p53, LOAEL

## Abstract

The existence of thresholds for carcinogenic compounds is an important topic in toxicology and regulatory science. Traditionally, genotoxic carcinogens are thought to exhibit no thresholds. However, cellular defense mechanisms like DNA repair and apoptosis can neutralize low levels of genotoxic stress implying different *Points of Departure* (PoDs) for different cellular endpoints. Moreover, since cellular PoDs are regulated by the DNA damage response (DDR) and the associated DNA damage signaling cascades, the question arises whether the DDR and its cellular outcome change, depending on the level of DNA damage. Here we analyzed whether PoDs for distinct cellular processes induced by benzo[a]pyrene-9,10-diol-7,8-epoxide (BPDE) are observed at the same or different level of DNA damage and whether these PoDs correlate with activation of different DNA damage signaling routes. BPDE represents the active metabolite of the polycyclic aromatic hydrocarbon benzo[a]pyrene (B[a]P) which is a product of incomplete combustion and therefore ubiquitously present in the natural environment. Our data indicate a PoD with a LOAEL (lowest observed adverse effect level) between 0.1 and 0.25 µM for DNA strand break formation, DDR activation, induction of cell death and cellular senescence. A high amount of cell death was observed at a dose of 1 µM and was accompanied by accumulation of DNA strand breaks and mediated by a switch from the p53^Ser15^ signaling axis to the p53^Ser46^ axis of the DDR. Importantly, BPDE-induced mutagenicity was observed predominantly at low BPDE concentration that failed to trigger the DDR and cellular senescence. These results suggest that low BPDE concentrations, which are unable to activate the DDR, are especially harmful in relation to mutation formation and carcinogenesis, eventually even more than DDR-activating concentrations.

**Supplementary Information:**

The online version contains supplementary material available at 10.1007/s00204-026-04426-8.

## Introduction

The existence of thresholds for carcinogenic compounds is a matter of ongoing debate in toxicology and regulatory science. Regarding toxic chemicals or other harmful genotoxins, such as ionizing radiation (IR), thresholds are defined as the maximum dose at which no toxicity occurs (Aldridge 1995; Purchase and Auton 1995). Carcinogens can be divided into two broad categories, genotoxic carcinogens, which directly damage DNA, potentially triggering mutations that lead to cancer, and non-genotoxic carcinogens, which cause cancer through other mechanisms, such as hormonal disruption, chronic inflammation, or sustained cell injury and regeneration (Bevan and Harrison 2017). Traditionally, genotoxic carcinogens have been assumed to follow a *linear no-threshold* (LNT) model concerning carcinogenesis. In this case, even the smallest dose could theoretically increase cancer risk without a safe exposure level. However, recent studies have shown that cellular defense mechanisms like DNA repair, apoptosis, and detoxification can neutralize low levels of genotoxic stress. These findings suggest the possibility of “practical thresholds” below which genotoxic agents may not cause harm. Therefore, *mode-of-action* (MoA)*-based thresholds* for certain genotoxic carcinogens, especially when the genotoxicity is indirect or secondary to other effects, is under debate. Moreover, agencies have incorporated the *Threshold of Toxicological Concern* (TTC) concept into guidelines. The TTC describes a level of exposure for defined classes of chemicals under which chronic exposure is assumed to be without appreciable risk over a lifetime. According the EFSA guidance on applying TTC in food safety, the TTC for genotoxic carcinogens is 0.0025 µg/kg bw/day (Committee et al. 2019). In line with this, the US FDA implemented the *Threshold of Regulation* (TOR) for substances migrating from food-contact materials, using a thresholds of 1.5 µg/person/day (~ 0.025 µg/kg bw/day) when toxicity data is lacking (Canady et al. 2013). Finally, within the ICH M7 guideline “*Assessment and Control of DNA Reactive (Mutagenic) Impurities in Pharmaceuticals to Limit Potential Carcinogenic Risk*” the TTC concept is used if there’s strong evidence of a threshold and low carcinogenic risk. Here, a daily intake of a mutagenic impurity of 1.5 µg is considered to be associated with a negligible risk (theoretical excess cancer risk of < 1 in 100,000 over a lifetime of exposure).

However, identification of thresholds for carcinogenesis is quite difficult. Given a low threshold, an unreasonable huge number of animals are needed to prove or disprove such a threshold. Moreover, even this does not imply that the same holds true in humans. Much easier is the experimental determination of a *Point of Departure* (PoD) for different cellular endpoints. Per definition, a PoD is the dose at which toxicity begins to manifest and can be expressed as NOAEL, LOAEL, BMD, or RfD. PoDs have already been discussed at the level of DNA adducts and repair mechanisms via gene mutations to tumorigenesis and are nevertheless not fully elucidated. What is known so far is that cells from different tissues are likely to have different PoDs with respect to DNA repair induction and efficacy (Thomas 2020; Thomas et al. 2015).

Cellular PoDs are most likely regulated by the DNA damage response (DDR). Important components of the DDR are the phosphatidylinositol-3-kinase (PI3K) like kinases ATM (ataxia telangiectasia mutated) and ATR (ATM and Rad3 related) (Ciccia and Elledge 2010; Harper and Elledge 2007; Lempiainen and Halazonetis 2009)). While ATM preferentially binds to DSBs, ATR responds to replication blockage and thus to single-stranded DNA (ssDNA) (Shiloh 2003). Subsequently, ATM phosphorylates CHK2 at Thr68 (Ahn et al. 2000) while ATR phosphorylates CHK1 at Ser317 and Ser345 (Niida et al. 2007). In turn, activated CHK1 and CHK2 function as p53^Ser20^ kinases (Shieh et al. 2000), thereby enhancing its tetramerization, stability, and activity. Additionally, ATM and ATR can phosphorylate p53 at Ser15 directly (Delia et al. 2000; Tibbetts et al. 1999). Phosphorylation of p53 at Ser15 as well as at Ser20 activates and stabilizes p53 (Saito et al. 2002) thereby increasing its transactivation activity (Chehab et al. 1999) and is mediated within minutes by DNA damage induction (Shieh et al. 1999). Moreover, ATM has been found to phosphorylate p53 at Ser46 upon IR, which is important for its apoptotic activity (Kodama et al. 2010; Saito et al. 2002). This phosphorylation allows recruitment of histone acetylase p300 which acetylates p53 at lysine 382 (Lys382) (Hofmann et al. 2002) altering its binding prevalence. In response, p53^Ser46^ preferentially activates pro-apoptotic factors. Besides ATM, an important kinase involved in phosphorylation of p53 at Ser46 is the Homeodomain-interacting protein kinase 2 (HIPK2) (D’Orazi et al. 2002; Sombroek and Hofmann 2009) which represents a nuclear serine/threonine kinase that acts as a corepressor for transcription factors (Kim et al. 1998). In unstressed cells, HIPK2 interacts with the E3 ubiquitin ligase “seven in absentia homolog-1” (SIAH1) which mediates HIPK2 polyubiquitination at numerous lysine residues leading to degradation and thereby inactivation of the kinase (Sombroek and Hofmann 2009; Winter et al. 2008). HIPK2 can be activated by several types of genotoxic damage (D’Orazi et al. 2002; Dauth et al. 2007; Di Stefano et al. 2004; Wesierska-Gadek et al. 2007). Thus, upon DNA damage, ATM/ATR- dependent phosphorylation of SIAH1 at Ser19 triggers the disruption of the HIPK2-SIAH1 complex resulting in HIPK2 stabilization and activation (Winter et al. 2008).

Activation of the DDR can have protective and cell-killing consequences. Importantly, it is not clear to which extent the DDR and its outcome change, depending on the level of DNA damage, and whether DDR signaling cascades switch above a given threshold, or if a fluent transition between the pathways exists. A critical question related to this topic is also, whether differential level of DNA damage trigger differential activation of the DDR and thereby different outcome-specific PoDs might exist.

To answer this question, we utilized benzo(a)pyrene 9,10-diol-7,8-epoxide (BPDE), the active metabolite of the polycyclic aromatic hydrocarbon benzo(a)pyrene (B[a]P) (Borgen et al. 1973; Huberman et al. 1976; Newbold and Brookes 1976; Slaga et al. 1976). B[a]P is a main carcinogen induced by incomplete combustion and is released into the atmosphere from industrial production processes and vehicle exhaust emissions and is produced during cigarette smoking and the cooking process. B[a]P has been shown to possess cytotoxic and tumor-promoting activity (Landolph et al. 1976; Reznik-Schuller 1975; Van Duuren and Goldschmidt 1976). Most importantly, B[a]P is also genotoxic and hence tumor initiating (Bukowska et al. 2022; Shah et al. 2016) and is therefore linked to a significantly increased risk of lung cancer. Moreover, B[a]P has been found to be associated with the development of the breast, esophagus, larynx, mouth, throat, kidney, bladder, pancreas, stomach, cervix, and blood cancer. Therefore, already in 1987, B[a]P was classified as a “probable human carcinogen”. In 2014, the Environmental Protection Agency (EPA) published a revised health assessment of B[a]P, upgrading its cancer classification to “carcinogenic to humans” (Baan et al. 2009). B[a]P itself is not carcinogenic unless metabolically activated by cytochrome P450, an epoxide hydrolase, to the ultimate carcinogen BPDE (Alexandrov et al. 2010). BPDE binds via the epoxide group to the exocyclic *N*^2^ position of guanine, thus forming bulky adducts in the DNA (Perlow et al. 2002), which can be repaired by nucleotide excision repair (NER) (Hess et al. 1997).

Here we determined PoDs for formation of strand breaks, cell death and cellular senescence, as well as mutation frequency and compared them with activation of DDR. Moreover, we analyzed how the DDR changes upon low and high concentrations. Our data indicate that for all cellular endpoints (except for mutations) a PoD was observed with a LOAEL of 0.1–0.25 µM. Cellular senescence was only observed within a narrow dose-window between 0.1 and 0.75 µM BPDE. Exceeding this dose, the DDR switched from a protective to a cell-killing response. On molecular level, the ATR/CHK1/p53^Ser15^ axis mediated activation of the NER and cellular senescence, whereas the ATM/CHK2/p53^Ser46^ axis induced induction of *NOXA* and cell death.

Of note, for mutation frequency, no threshold was observed. Importantly, at concentrations above 0.25 µM, the induction of cellular senescence prevented mutations, indicating that low concentrations which are unable to induce the DDR and thereby cellular senescence might be more harmful for a multicellular organism than DDR-activating concentrations.

## Material and methods

### Cell culture, drug treatment, siRNA-mediated knockdown and pharmacological inhibition

The human diploid VH10tert foreskin fibroblast cell line was immortalized by stable transfection with the telomerase gene (TERT) and kindly provided by Prof. L. Mullenders (Department of Toxicogenetics, Leiden University Medical Centre, the Netherlands) and cultivated in Dulbecco’s minimal essential medium (DMEM) containing 10% FCS and 1% L-glutamine under normal atmosphere (5% CO_2_) at 37 °C. Cells were regularly checked for mycoplasma contamination.

Activated r-7,t-8-Dihydroxy-t-9,10-epoxy-7,8,9,10-tetrahydrobenzo[a]pyrene (anti-BPDE; CAS no. 58917-67-2) was synthesized from trans-7,8-dihydroxy-7,8-dihydrobenzo(a)pyrene (Platt and Oesch 1983) by Dr. A. Seidel as described (Yagi et al. 1977).

For gene silencing, pre-designed ATM- and CHK2-specific siRNAs were used (Ambion), the HIPK2-specific siRNA was custom-made (5’-CGGACUCACCAUAUCCUUUdTdT-3’). The human non-silencing (ns)-siRNA (Silencer Select Predesigned siRNA Negative Control #1 siRNA, Ambion) was used as negative control. For siRNA transfection, the Lipofectamine® RNAiMAX Transfection Reagent (13,778,075, Invitrogen) and Opti-MEM™ Reduced Serum Medium (Gibco) were used. The ATM inhibitor KU-60019 (S1570, Selleck Chemicals Llc), the ATR inhibitor VE-821 (S8007, Selleck Chemicals Llc), the CHK2 inhibitor Chk2 Inhibitor II hydrate (C3742, Sigma Aldrich), and the homologous recombination (HR) inhibitor RAD51 inhibitor RI-1 (415,713-60-9, Axon Medchem BV) were used at 5 µM. The Caspase-6 Inhibitor I (21,875, Millipore) was used at 20 µM and the CHK1 inhibitor UCN-01 (U6508, Sigma Aldrich) at 100 nM.

### Determination of clonogenic survival

For determining clonogenic survival via colony formation assay, 1000 cells were seeded per 6-cm dish and treated 6 h afterwards with BPDE. Two weeks later, the grown colonies were fixed, stained, counted and put into relation to the untreated control, which was set at 100%. Experiments were performed in biological triplicates.

### Determination of cell cycle progression, cell death, and senescence

For analysis of cell cycle distribution, cells were stained with propidium iodide (PI) followed by the determination of the DNA content via flow cytometry using BD FACSCanto™ II. To quantify apoptosis induction, cells were double-stained with PI and Annexin V and analyzed by flow cytometry. Experiments were performed in biological triplicates and 10,000 cells were analyzed in each experiment. Senescence induction was assessed microscopically using the β-galactosidase (β-Gal) assay as described before (Aasland et al. 2019; Sallbach et al. 2024). Experiments were performed in biological triplicates and 500 cells were analyzed in each experiment. The MTT assay was performed as described (Tomicic et al. 2014). Experiments were performed in biological triplicates and in each experiment, 10,000 cells were seeded in 6 wells of a 96-well plate for each concentration.

### HPRT mutagenicity assay

For determination of the mutation frequency, hypoxanthine–guanine phosphoribosyltransferase (HPRT) assay was performed as described (Christmann et al. 2016). In brief, VH10tert cells were incubated with BPDE and seven days later, 1000 cells per experimental point were re-seeded in triplicates and the relative plating efficiency was determined using the clonogenic survival assay in non-selective medium. For the determination of mutation frequency, the remaining cells were re-seeded at a concentration of 1 × 10^7^ in selective medium (6-thioguanine, 2.5 μg/ml, Sigma). Three weeks later, colonies were fixed, stained and counted. Mutation frequency was determined by correcting mutant colony counts for plating efficiency. Experiments were performed in biological quadruplicates.

### Preparation of protein extracts and western blot analysis

For preparing whole-cell protein extracts, cells were washed with PBS, harvested, and re-suspended in Net-N lysis buffer (10 mM Tris–HCl, pH 8.0, 1 mM EDTA, 712.5 mM NaCl, 9.95% glycerin, 0.2% NP-40, 1% Phosphatase Inhibitor Cocktail 2 (P5726, Sigma), 14.25% Protease inhibitor Complete™ (Roche)). After 25 min incubation on ice, the samples were sonicated and centrifuged. The protein content in the supernatant was determined using Bradford protein quantification assay. Specific mouse and rabbit mAb used for protein detection, as well as secondary antibodies are listed in the Suppl. Table 1. Pierce™ ECL Western Blotting Substrate (Thermo Fisher) and the iBright™ CL1000 were used to visualize the protein-antibody complexes. To verify the size of the detected proteins, the Spectra™ Multicolor Broad Range Protein Ladder (26,634, Thermo Fisher) was used as a reference.

### Preparation of RNA, cDNA, and real-time qPCR

Total RNA was isolated using the NucleoSpin® RNA Kit (740,955, Machery-Nagel). cDNA was synthesized from 1 µg RNA using the Verso cDNA Synthesis Kit (AB1453A, Thermo Fisher). qPCR was performed in technical triplicates using the GoTaq® qPCR Master Mix Protocol (A6001, Promega) and the CFX384™ Real-Time System (Biorad) for detection. Non-template controls were included, and specific primers are listed in the Suppl. Table 1. For evaluation, the CFX Manager™ software (Biorad) was used, expression was normalized to *ACTB* and *GAPDH*, and the untreated control was set to one. Experiments were performed in technical triplicates.

### Immunofluorescence (IF) staining

Cells were seeded, treated, and incubated on etched cover slips in 6-well plates. For fixation, cells were first washed with PBS and then incubated for 8 min at − 20 °C with ice-cold methanol-acetone (7:3) fixation solution. Afterwards, cover slips were washed with PBS and incubated for 2 h at RT with 5% BSA, PBS for blocking. Primary antibody solution (1:400 in 5% BSA, PBS) was applied and overnight incubation took place at 4 °C in a humidity chamber. Cover slips were then washed briefly with PBS, covered with 0.25% Triton X100, PBS for 10 min, and washed again three times with PBS. Then, the secondary antibody solution (1:400 in 5% BSA, PBS) mixed with DAPI (1:1000) was applied for 2 h at RT in the dark. After three final washing steps in PBS, the cover slips were stained again with DAPI (1:1000 in 5% BSA, PBS) and without additional washing mounted with Vectashield mounting medium containing DAPI. Microscopical analyses were performed using Zeiss Axio Imager M1 fluorescence microscope (Carl Zeiss AG) and the Metafer 4 V3.5.0 software (MetaSystems GmbH). Antibodies are listed in the Suppl. Table 1. Experiments were performed in biological triplicates and 500 cells were analyzed in each experiment.

### Comet assay

For neutral and alkaline comet assay, cells were washed with PBS, harvested, and re-suspended in ice-cold PBS to a final concentration of 40,000 cells per 120 µl PBS. Then, 120 µl cell suspension and 120 µl 1%-low gelling temperature (LGT) agarose were mixed and 120 µl of the cell agarose mixture were applied to microscope slides pre-coated with 1.2% agarose. Embedded cells were then lysed for 1 h at 4 °C either in neutral (pH 7.5) or alkaline (pH 10.0) lysis buffer (2.5 M NaCl, 0.1 M EDTA disodium, 9.9 mM Tris, 1% Triton X100). For neutral comet assay, neutral electrophoresis buffer (90.2 mM Tris, 89.9 mM boric acid, 2 mM EDTA disodium, pH 7.5) was used and electrophoresis was run for 25 min at 25 V. Afterwards, microscope slides were briefly immersed in dH_2_O and then fixed with ice-cold methanol for 10 min at − 20 °C and air dried overnight. For alkaline comet assay, alkaline electrophoresis buffer (0.3 M NaOH, 1 mM EDTA disodium, pH > 13) was used. Microscope slides were placed in the electrophoresis buffer to allow unwinding of the DNA for 20 min in the dark and electrophoresis was run afterwards for 15 min at 300 mA. Subsequently, microscope slides were immediately immersed for 5 min in neutralization buffer (0.4 M Tris, pH 7.5), fixed with ice-cold methanol for 10 min at − 20 °C, and air dried overnight. For analysis of strand break induction by measuring the Olive tail moment, DNA was stained with PI, and 50 cells per microscope slide were evaluated using Comet Assay IV 4.3 (Perceptive Instruments Ltd). Experiments were performed in biological triplicates.

### Quantification and statistical analysis

For quantification and statistical analyses, GraphPad Prism version 8.02 for Windows (GraphPad Software, La Jolla, California, USA), was utilized. For pair wise comparisons, Student’s *t*-test was used for data evaluation. For evaluation of dose-kinetics, One-way or Two-way ANOVA with Dunnett correction was performed. Data were expressed as a mean ± SD. Hereby, statistical significance was defined as *p ≤ 0.05 (significant), **p ≤ 0.01 (very significant), ***p ≤ 0.001 (highly significant), ****p ≤ 0.0001 (extremely significant).

## Results

### The DDR mediates opposite cellular fates at low and high BPDE concentrations

**Fig. 1:**
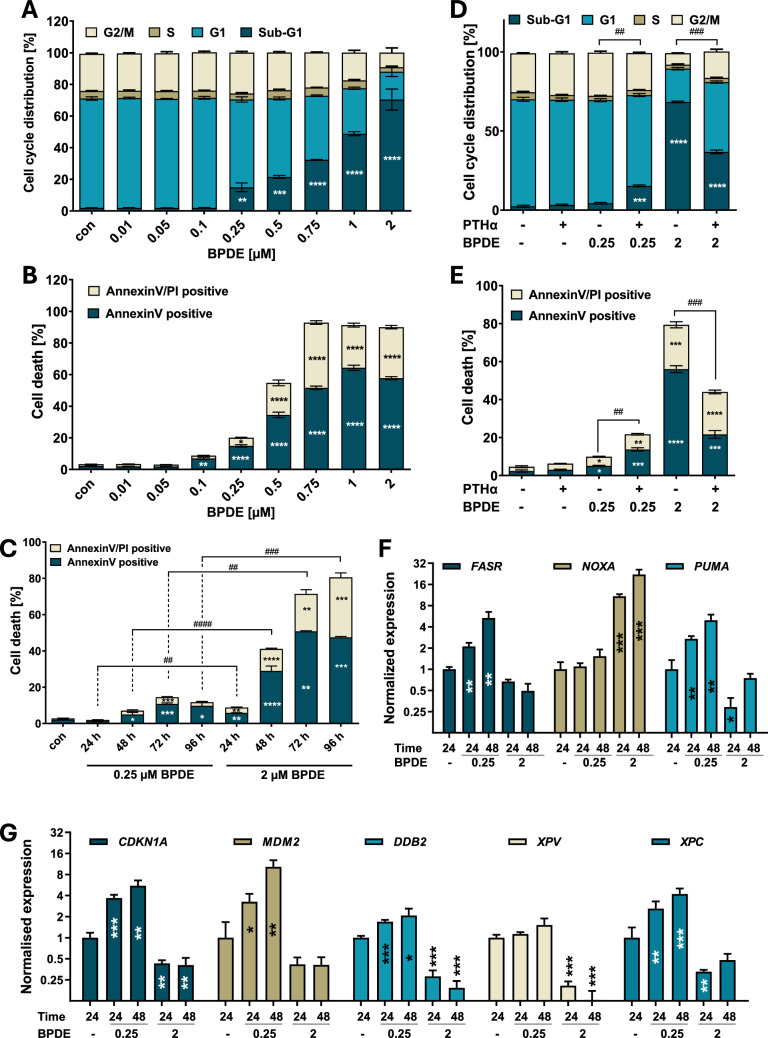
Time- and dose-dependent induction of cell death upon BPDE. **A** Cell cycle distribution and cell death induction were assessed 96 h after BPDE treatment in VH10tert cells via flow cytometry using PI staining. One-way ANOVA with Dunnett correction was performed to compare the SubG1 fraction in BPDE-exposed and non-exposed (con) cells. **B** Dose-dependent induction of apoptosis and necrosis was analyzed via flow cytometry using Annexin V/PI staining 96 h after BPDE treatment. Two-way ANOVA with Dunnett correction was performed to compare BPDE-exposed and non-exposed (con) cells. **C** Time-dependent induction of apoptosis and necrosis was analyzed via flow cytometry using Annexin V/PI staining after treatment with 0.25 or 2 µM BPDE (*). Two-way ANOVA with Dunnett correction was performed to compare BPDE-exposed and non-exposed (con) cells (*). Pairwise comparison between 0.25 and 2 µM treated cells at the same time point was statistically analyzed using Student’s *t* test (^#^). **D**/**E** Cells were treated with PTHα 1 h prior to BPDE exposure. Cell cycle distribution and cell death induction was analyzed after 96 h via flow cytometry using PI staining **D** and induction of apoptosis and necrosis was analyzed via flow cytometry using Annexin V/PI staining **E**. Pairwise comparison between PTHα and/or BPDE-exposed with non-exposed cells was statistically analyzed using Student’s *t* test (*). Pairwise comparison between PTHα-pretreated with non-pretreated cells upon exposure to 2.5 and 2 µM BPDE was statistically analyzed using Student’s *t* test (^#^). **A**–**E** Experiments were performed in three biological replicates and 10,000 cells were analyzed in each experiment. **F**/**G** mRNA expression of p53-target genes was assessed in VH10tert cells 24 and 48 h after BPDE treatment via qPCR; *ACTB* and *GAPDH* were used as internal loading control. Experiments were performed in technical triplicates. Pairwise comparison between BPDE-exposed and non-exposed cells was statistically analyzed using Student’s *t* test. **A**–**G** not labeled = not significant, */^#^*p* ≤ 0.05, **/^##^*p* ≤ 0.01, ***/^###^*p* ≤ 0.001, ****/^#####^*p* ≤ 0.0001)

To determine to which extent activation of DDR and cell fate alter, depending on the concentration, and to identify potential threshold in vitro, human non-transformed fibroblasts (VH10tert) were exposed to the environmental carcinogen BPDE. First, the dose-dependent induction of cell death (Sub-G1) and cell cycle distribution were analyzed by PI staining. The results indicate a low but significant number of dead cells at a concentration of 0.25 µM BPDE (~ 15%), which increased up to ~ 70% upon exposure to 2 µM BPDE (Fig. 1A). Concentrations below 0.25 µM BPDE showed no cytotoxicity. Similar results were observed detecting apoptotic (Annexin V positive) and necrotic (Annexin V/PI positive) cells. Here, 0.25 µM BPDE induced ~ 15% apoptotic and ~ 5% necrotic cells, which increased up to ~ 65% (apoptotic) and ~ 25% (necrotic) cells upon exposure to 2 µM BPDE (Fig. 1B). In this assay, significant formation of apoptotic cells was observed already at 0.1 µM. To show linearity of the dose-dependent cell death upon BPDE exposure, alternative plotting of the data and linear regression is presented in Suppl. Fig. 1A/B.

We decided to use 0.25 and 2 µM BPDE to compare the molecular consequences and cellular outcome of low-dose and high-dose exposure. First, time-dependent measurement of apoptotic and necrotic cells was performed to ensure that upon 0.25 µM BPDE, toxicity was also not observed at later periods. Indeed, in a time-window between 24 and 96 h, cell death never exceeded 15% (Fig. 1C). In contrast, after 96 h nearly all cells entered apoptosis or necrosis upon exposure to 2 µM BPDE.

To determine the impact of the DDR in cell death, p53 was inhibited using pifithrin α (PTHα) and cells were exposed to 0.25 and 2 µM BPDE. Both, detection of Sub-G1 (Fig. 1D) and of apoptotic/necrotic cells (Fig. 1E) revealed that p53 inhibition sensitized cells against 0.25 µM and protected against 2 µM BPDE, indicating a switch from pro-survival to pro-death effects of the DDR, i.e. of the p53 activity in this dose range.

To analyze the molecular mechanisms underlying the switch from pro-survival to pro-death effects of p53, the expression of p53 target genes was analyzed by RT-qPCR. Exposure to 0.25 µM transcriptionally activated the pro-apoptotic factors *FASR* and *PUMA*, as well as the protective factors *CDKN1A* (p21), *MDM2*, *DDB2*, and *XPC*. *XPV* (PolH) was only very weakly activated. Upon exposure to 2 µM BPDE, the expression of all these factors was strongly repressed (Fig. 1F/G). In contrast, 2 µM BPDE exclusively activated the pro-apoptotic factor *NOXA* (Fig. 1F). Dose-dependent differences were also observed for anti-apoptotic factors. Here, exposure to 0.25 µM transcriptionally activated *BCL-xL* and *c-IAP1*, whereas upon exposure to 2 µM BPDE, the expression was repressed (Suppl. Fig. 1C). Opposite results were observed for Survivin (*BIRC5*), which was repressed at 0.25 and induced at 2 µM BPDE. Of note, Survivin (*BIRC5*) is known to be transcriptionally repressed by p53. The expression of *XIAP* was only slightly reduced (Suppl. Fig. 1C).

**Fig. 2:**
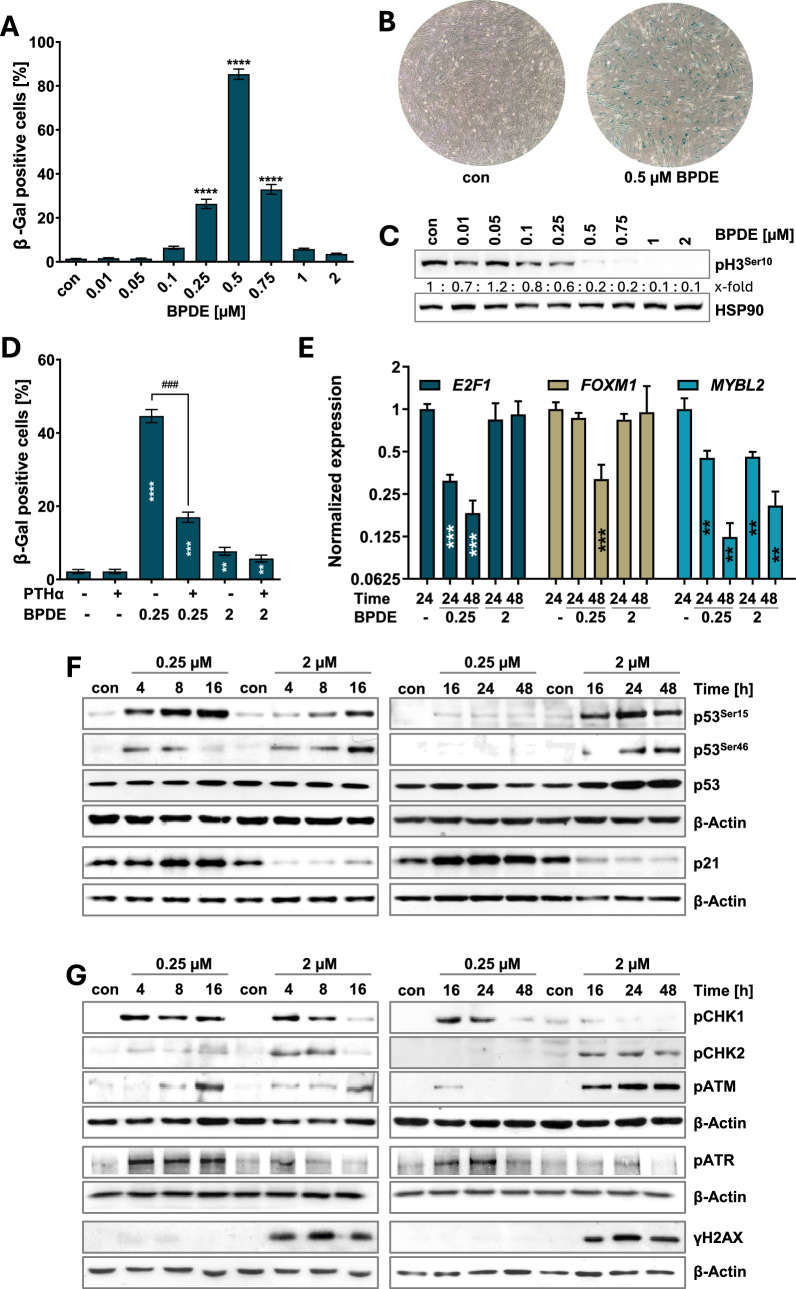
Time- and dose-dependent induction of senescence and DDR upon BPDE. **A** Cellular senescence was assessed 120 h after BPDE treatment of VH10tert cells via β-Gal staining. Experiments were performed in biological triplicates, counting 500 cells in each experiment. One-way ANOVA with Dunnett correction was performed to compare BPDE-exposed with non-exposed (con) cells. **B** Representative image of senescent cells. **C** Expression of pH3^Ser10^ protein was measured 120 h after BPDE treatment via immunodetection; HSP90 was used as internal loading control. **D** VH10tert cells were treated with PTHα 1 h prior to BPDE exposure and cellular senescence was assessed 120 h after BPDE treatment of VH10tert cells via β-Gal staining. Experiments were performed in biological triplicates, counting 500 cells in each experiment. Pairwise comparison between PTHα and/or BPDE-exposed with non-exposed cells was statistically analyzed using Student’s *t* test (*). Pairwise comparison between PTHα-pretreated and non-pretreated cells upon exposure to 0.25 and 2 µM BPDE was statistically analyzed using Student’s *t* test (^#^). **E** mRNA expression of p53-target genes was assessed in VH10tert cells 24 and 48 h after BPDE treatment via qPCR; *ACTB* and *GAPDH* were used as internal loading control. Experiments were performed in technical triplicates. Pairwise comparison between BPDE-exposed and non-exposed cells was statistically analyzed using Student’s *t* test. **F** Expression and phosphorylation of p53, as well as expression of p21 were measured at different time points after treatment with 0.25 and 2 µM BPDE via immunodetection; β-Actin was used as internal loading control. **G** Expression and phosphorylation of CHK1, CHK2, ATR, ATM, and H2AX was measured at different time points after 0.25 and 2 µM BPDE treatment via immunodetection; β-Actin was used as internal loading control. **A**/**D**/**E** not labeled = not significant, */^#^*p* ≤ 0.05, **/^##^*p* ≤ 0.01, ***/^###^*p* ≤ 0.001, ****/^#####^*p* ≤ 0.0001)

Another outcome of genotoxic stress is cellular senescence. Therefore, the dose-dependent activation of senescence was analyzed using the SA-β-Gal assay. Interestingly, our analysis revealed that senescent cells were observed in a narrow dose range between 0.1 and 1 µM, reaching its maximum with ~ 90% at 0.5 µM BPDE (Fig. 2A/B and Suppl. Figure 2 for representative images). To show that the dose-dependent senescence upon BPDE exposure is non-linear, alternative plotting of the data and linear regression is presented in Suppl. Fig. 1D. At higher concentrations, both, a lack in senescent cells and in overall cell count was observed, presumably due to cell death. Abrogation of proliferation was also observed using detection of histone 3 phosphorylated at Ser10 (H3^Ser10^), which represents a marker for mitosis. Starting at 0.25 µM, a strong decrease of H3^Ser10^ was found (Fig. 2C and Suppl. Fig. 3A for additional experiments). Again, the impact of p53 was analyzed using PTHα, showing that senescence was p53-dependent (Fig. 2D and Suppl. Fig. 3B for representative images). Since we previously showed that senescence is mediated by p21-dependent DREAM activation (Schmidt et al. 2024), we compared the expression of the essential DREAM targets and cell cycle regulators *E2F1*, *FOXM1* and *MYBL2* (B-MyB) by qPCR (Fig. 2E). Unlike p53 targets, the DREAM targets *E2F1* and *FOXM1* were repressed upon 0.25 but not upon 2 µM BPDE. Both BPDE concentrations repressed *MYBL2*. The missing repression of *E2F1* and *FOXM1* at higher BPDE concentrations indicates that the DREAM complex is only activated at low concentrations of BPDE, reflecting the differential regulation of p21. An important feature of senescent cells is the activation of the senescence-associated secretory phenotype (SASP) (Coppe et al. 2010). Indeed, upon exposure to 0.25 µM BPDE, the cells responded with induction of IL-6 and IL-8 (Suppl. Fig. 1E).

### Different DDR signaling routes are active at low versus high BPDE concentrations

Differential p53 phosphorylation, in particular phosphorylation of Serine 46 has been linked to cell fate switch towards apoptosis upon DNA damage (Liebl and Hofmann 2019). To analyze the molecular mechanism underlying the different cell fate outcomes, activation/phosphorylation of p53 was analyzed via immunodetection (Fig. 2F for a representative experiment and Suppl. Fig. 4A for its densitometrical evaluation). A second experiment and densitometrical evaluation is presented in Suppl. Fig. 5. Activation of p53 was already observed at early time points after BPDE exposure, and prominent phosphorylation of p53 at Ser15 was observed already 4 h after exposure to 0.25 µM BPDE. Interestingly, at these early time points, weaker Ser15 phosphorylation was observed upon 2 µM BPDE. However, at later time points (24 and 48 h) the p53^Ser15^ phosphorylation was exclusively observed at high BPDE concentrations. p53^Ser15^ is known to be responsible for the transcriptional activity of p53 and thereby activation of its target genes (see Fig. 1F/G). In contrast, phosphorylation of p53 at Ser46 is associated with induction of a small subset of pro-apoptotic p53 targets, such as *NOXA* (Ichwan et al. 2006). Weak phosphorylation of p53 at Ser46 was observed at early time points upon low and high BPDE concentrations, whereas at late time points phosphorylation was exclusively found upon 2 µM BPDE. We should note that the early (con-16) and late (con-48) experiments were performed independently, explaining the differing results at 16 h. Similar to its transcriptional regulation, the induction of the main p53 target p21 was exclusively observed at low but not at high BPDE concentrations. Obviously, and in line with previous reports (Ichwan et al. 2006), phosphorylation of p53 at Ser46 is linked to the pro-apoptotic p53 response and interferes with the induction of other p53 targets.

Next, we studied the activation of the main signaling components of the DNA damage signaling pathway, including the checkpoint kinases ATM, ATR, CHK1 and CHK2. Again, clear differences in the concentration-dependent response to BPDE exposure were observed (Fig. 2G for a representative experiment and Suppl. Fig. 4B for its densitometrical evaluation). A second experiment and densitometrical evaluation is presented in Suppl. Fig. 6. Phosphorylation of ATR and CHK1 was detected between 4 and 24 h upon low concentrations, whereas it was only observed between 4 and 8 h upon high concentrations. In contrast, phosphorylation of CHK2 was mainly detected upon high concentrations and ATM was transiently phosphorylated 16 h upon low concentrations, whereas its phosphorylation was strongly activated 16–48 h after exposure to high concentrations. This suggests that low concentrations of BPDE predominantly induce ATR-related replicative stress, which is transient and resolved 16 h after exposure. In contrast, upon higher BPDE concentrations, these breaks seem not to be repaired and therefore strongly activate the ATM-CHK2 axis.

### Altered DDR leads to differential phosphorylation of p53 at low and high BPDE concentrations

The observed differential phosphorylation of p53 might be responsible for the observed cellular responses upon low and high BPDE concentrations. Therefore, we analyzed the molecular mechanisms underlying p53 phosphorylation. The main kinase involved in phosphorylation of p53 at Ser46 is the Homeodomain-interacting protein kinase 2 (HIPK2) (D’Orazi et al. 2002; Sombroek and Hofmann 2009) which represents a nuclear serine/threonine kinase that acts as a corepressor for transcription factors (Kim et al. 1998).

**Fig. 3:**
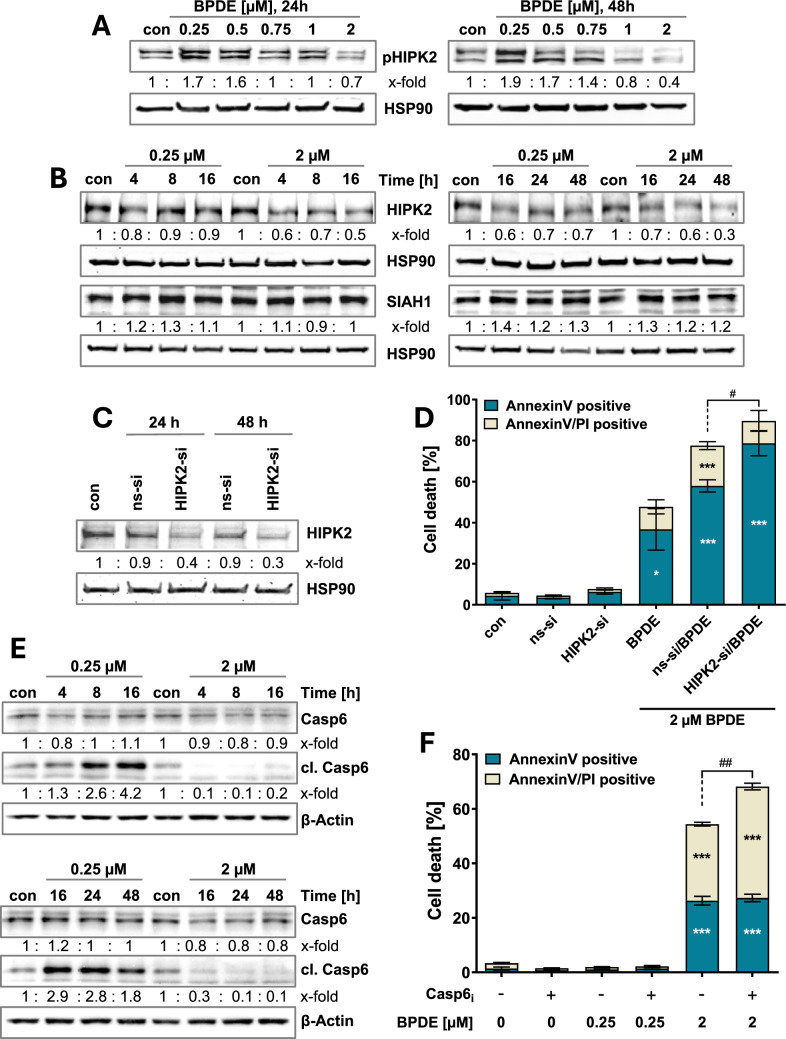
Impact of HIPK2 on BPDE-induced cell death. **A** Phosphorylation of HIPK2 was measured 24 and 48 h after BPDE exposure (0.25 – 2 µM) via immunodetection; HSP90 was used as internal loading control. **B** Expression of HIPK2 and SIAH1 was measured at different time points upon 0.25 and 2 µM BPDE via immunodetection; HSP90 was used as internal loading control. **C** VH10tert cells were transfected with ns- and HIPK2-siRNA. Downregulation of HIPK2 protein was assessed 24 and 48 h after transfection via immunodetection; HSP90 was used as internal loading control **D** VH10tert cells were transfected with ns- and HIPK2-siRNA and 24 h later exposed to BPDE. Cell death induction was analyzed 72 h later via flow cytometry using Annexin V/PI staining. Pairwise comparison between siRNA and/or BPDE-exposed versus non-exposed cells (con) was statistically analyzed using Student’s *t* test (*). Pairwise comparison between ns-siRNA and HIPK2-siRNA pretreated cells upon BPDE exposure was statistically analyzed using Student’s *t* test (^#^). **E** Expression of full-length and cleaved caspase-6 was determined at different time points after 0.25 and 2 µM BPDE treatment via immunodetection; β-Actin was used as internal loading control. **F** VH10tert cells were pre-treated with a caspase-6 inhibitor and 1 h later exposed to BPDE. Cell death induction was analyzed 72 h after BPDE treatment via flow cytometry using Annexin V/PI staining. Pairwise comparison between caspase-6 inhibitor and/or BPDE-exposed versus non-exposed cells was statistically analyzed using Student’s *t* test (*). Pairwise comparison between caspase-6 inhibitor pretreated and non-pretreated cells upon BPDE exposure was statistically analyzed using Student’s *t* test (^#^). **D**/**F** Experiments were performed in biological triplicates and 10,000 cells were analyzed in each experiment. not labelled = not significant, */^#^*p* ≤ 0.05, **/^##^*p* ≤ 0.01, ***/^###^*p* ≤ 0.001, ****/^#####^*p* ≤ 0.0001)

Our data show that a weak phosphorylation of HIPK2 at Thr880/Ser882 was occured upon 0.25 and 0.5 µM BPDE; at toxic concentrations, a reduced phosphorylation was observed (Fig. 3A). Moreover, a reduced HIPK2 expression was detected upon toxic concentrations, whereas SIAH1 expression remained unaltered (Fig. 3B). HIPK2 can also be activated by caspase-mediated cleavage. Upon DNA damage, caspase-6 is transcriptionally activated by p53 (MacLachlan and El-Deiry 2002). Caspase-6 can cleave HIPK2 at Asp916 and Asp977, thereby removing the autoinhibitory domain of HIPK2, resulting in a catalytically hyperactive HIPK2 protein (Gresko et al. 2006; Sombroek and Hofmann 2009). Since a decreased expression of HIPK2 at high concentrations was observed, a potential activation of HIPK2 by caspase-6 was analyzed. However, upon BPDE exposure, the basal caspase-6 expression remained unaltered, independently of time and concentration, while its cleavage (activation) was only observed at low but not high concentrations (Fig. 3E). Besides the lack of activation at toxic concentrations, the biological impact of HIPK2 on BPDE-induced senescence was analyzed. Thus, transient downregulation of HIPK2 via siRNA enhanced toxicity upon 2 µM BPDE instead of reducing it (Fig. 3C/D). Interestingly, similar to HIPK2 inhibition, inhibition of caspase-6 activity also increased cell death induction upon 2 µM BPDE (Fig. 3F).

**Fig. 4:**
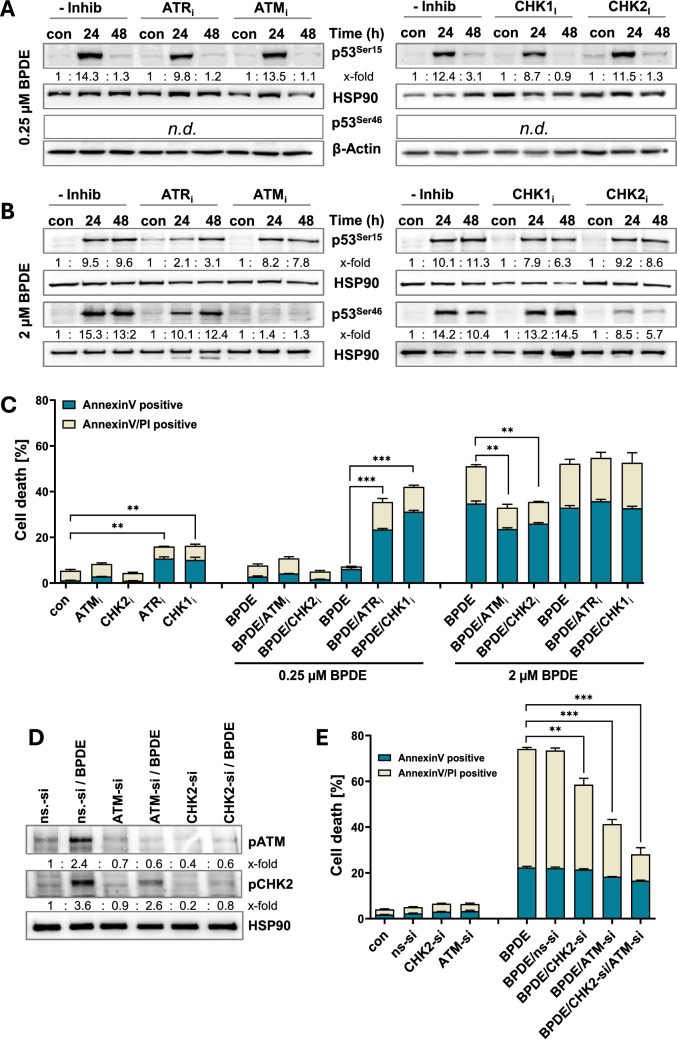
Impact of the DDR on BPDE-induced cell death. **A**/**B** VH10tert cells were treated with inhibitors against ATR, ATM, CHK1 and CHK2 1 h prior to exposure to 0.25 **A** or 2 µM **B** BPDE. Phosphorylation of p53 at serine 15 and serine 46 was assessed via immunodetection and HSP90 or β-Actin were used as internal loading control. **C** VH10tert cells were treated with inhibitors against ATR, ATM, CHK1, and CHK2 1 h prior to exposure to 0.25 or 2 µM BPDE. Cell death induction was analyzed 72 h after exposure via flow cytometry using Annexin V/PI staining. **D** VH10tert cells were transfected with ns-, ATM-, or CHK2-siRNA and treated with 2 µM BPDE 24 h later. Downregulation of pATM or pCHK2 was assessed 48 h after transfection via immunodetection; HSP90 was used as internal loading control. **E** VH10tert cells were transfected with ns-, ATM-, or CHK2-siRNA and 24 h later exposed to BPDE. Cell death induction was analyzed 72 h after BPDE treatment via flow cytometry using Annexin V/PI staining. **C**/**E** Experiments were performed in biological triplicates and 10,000 cells were analyzed in each experiment. Differences between combined apoptosis/necrosis induced by BPDE and BPDE/inhibitor treatment **C**, as well as differences between BPDE-exposed and BPDE/siRNA-exposed cells were statistically analyzed using Student’s *t* test (ns = not significant, *p ≤ 0.05, **p ≤ 0.01, ***p ≤ 0.001)

Since HIPK2 is not involved in Ser46 phosphorylation of p53 upon BPDE exposure, other p53 Ser46 kinases, such as ATM, must play a major role (Liebl and Hofmann 2019). To obtain deeper insight, ATR, ATM, CHK1 and CHK2 were inhibited before BPDE exposure and protein expression of p53^Ser15^ and p53^Ser46^ was investigated. At low BPDE concentrations, Ser46 phosphorylation was not detected (n.d.). Of note, neither inhibition of ATR, ATM, CHK1 or CHK2 was strongly preventing p53 Ser15 phosphorylation, suggesting that they can compensate for each other. (Fig. 4A for a representative experiment). A second experiment and densitometrical evaluation is presented in Suppl. Fig. 7. At high BPDE concentrations, ATR inhibition slightly reduced Ser15 and Ser46 phosphorylation, whereas inhibition of ATM fully blocked Ser46 phosphorylation and CHK2i slightly reduced it (Fig. 4B for a representative experiment). A second experiment and densitometrical evaluation is presented in Suppl. Fig. 7). In addition, the impact of kinase inhibition on apoptosis and necrosis induction was analyzed. Inhibition of ATR or CHK1 increased cell death rates in untreated cells and at low, but not at high BPDE concentrations (Fig. 4C). Opposed to this, ATM or CHK2 inhibition reduced cell death at high concentrations. Finally, also knockdown of ATM and CHK2 protected from cell death (Fig. 4D/E), indicating that ATM and CHK2 drive cell death via Ser46 phosphorylation of p53. We should state that in some experiments, the ATR inhibitor induced p53-Ser15 phosphorylation and ATR and CHK1 inhibition and weakly caused cell death by themself, which is most likely caused by replication stress induced by their inhibition in replicating cells.

### Threshold concentrations are defined by the repairability of the resulting DSBs

**Fig. 5:**
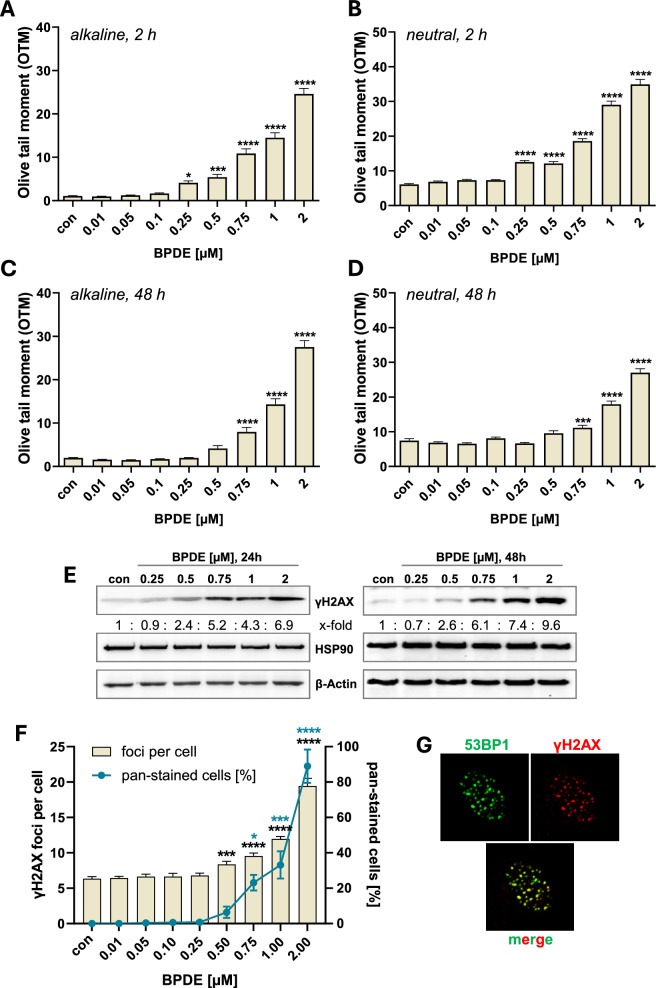
Time- and dose-dependent induction of single and double-strand breaks upon BPDE. **A**–**D** SSB and DSB induction upon BPDE was assessed in VH10tert cells using the alkaline **A**/**C** and neutral **B**/**D** comet assay 2 h **A**/**B** or 48 h **C**/**D** after BPDE exposure. *Comet IV* was used for the evaluation of the comet assay and the mean Olive tail moment is shown. **A**–**D** Experiments were performed in biological triplicates, analyzing 50 cells per experiment. One-way ANOVA with Dunnett correction was performed to compare BPDE-exposed and non-exposed cells (con). **E** Dose-dependent expression and phosphorylation of H2AX were measured 24 and 48 h after BPDE treatment via immunodetection; β-Actin was used as internal loading control. **F** Dose-dependent formation of γH2AX foci and presence of pan-stained cells were measured 48 h after BPDE exposure using the Metafer system and immunostaining. Experiments were performed in biological triplicates, analyzing 500 cells per experiment. One-way ANOVA with Dunnett correction was performed to compare BPDE-exposed and non-exposed cells (con). **G** Co-localization between γH2AX and 53BP1 was analyzed using laser scanning microscopy 48 h after BPDE exposure. **A**–**D**/**F** not labeled = not significant, *p ≤ 0.05, **p ≤ 0.01, ***p ≤ 0.001, ****p ≤ 0.0001)

The ATR-CHK1 axis is mediated predominantly via replicative stress (Petermann and Caldecott 2006) and the ATM-CHK2 axis via DSBs (Ismail et al. 2005). Therefore, formation of DSBs was measured after 2 and 48 h BPDE exposure using the comet assay. Whereas the neutral comet assay primarily detects DNA double-strand breaks, the alkaline comet assay detects single-strand breaks, alkali-labile sites (including AP sites), and double-strand breaks (He et al. 2025). Both assays revealed strand break formation starting at 0.25 µM BPDE after 2 h (Fig. 5A–D). After 48 h, significant strand break formation was observed starting at 0.75 µM BPDE, indicating saturation of DNA repair and persistence of the DNA strand breaks. To show that the dose-dependent strand formation upon BPDE exposure is linear, alternative plotting of the data and linear regression is presented in Suppl. Fig. 8A.

Another marker for DSBs is the phosphorylation of H2AX (Kuo and Yang 2008). Therefore, concentration-dependent phosphorylation of H2AX (γH2AX) was analyzed. Using immunodetection, H2AX phosphorylation was observed starting at 0.5 µM BPDE (Fig. 5E for a representative experiment and Suppl. Fig. 8B for an additional independent experiment, including densitometrical evaluation). Using immunofluorescence, significant H2AX phosphorylation was observed starting at 0.5 µM BPDE (Fig. 5F). In addition, immunofluorescence allows the quantitative measurement of individual DSBs, revealing up to 20 DSBs per cell upon 2 µM BPDE. However, this number might only represent the surviving cells since up to 90% of the cells showed pan-γH2AX staining, which can be taken as a marker for dying cells. As a proof that the γH2AX foci indeed represent DSBs, the co-localization of γH2AX with 53BP1 was analyzed (for representative picture, see Fig. 5G).

**Fig. 6:**
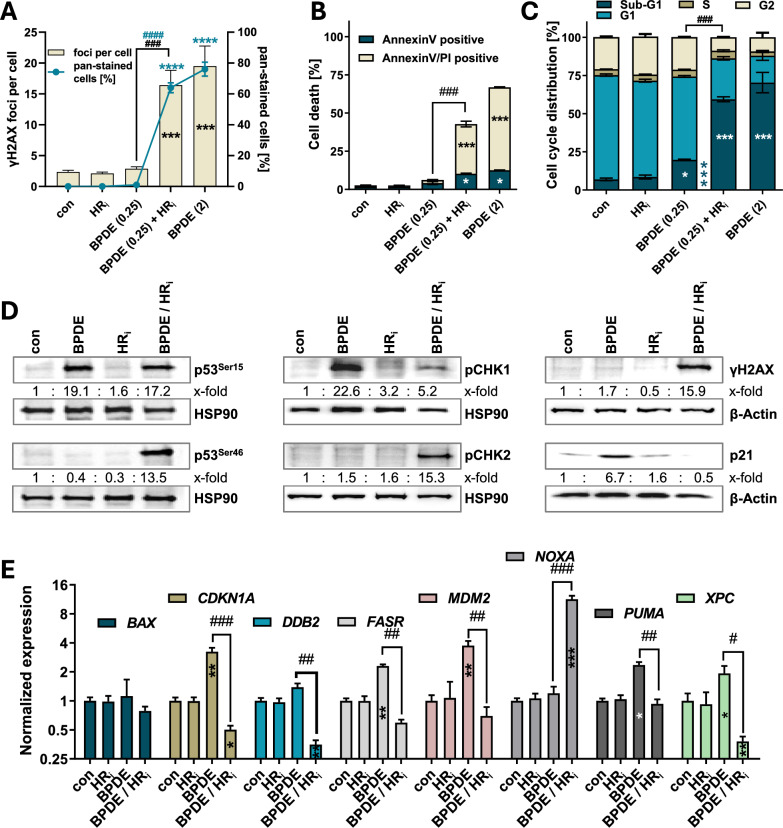
Impact of HR on BPDE-induced DDR and cell death. VH10tert cells were treated with an inhibitor against Rad51 (HR_i_) 1 h prior to exposure to 0.25 or 2 µM BPDE. **A** Dose-dependent formation of γH2AX foci and presence of pan-stained cells were measured 48 h after BPDE exposure by immunostaining. Experiments were performed in biological triplicates, analyzing 500 cells per experiment. **B** Cell death induction was analyzed 72 h after exposure via flow cytometry using Annexin V/PI staining. Experiments were performed in biological triplicates, analyzing 10,000 cells per experiment. **C** Cell cycle distribution and cell death induction were assessed 72 h after BPDE treatment of VH10tert cells via flow cytometry using PI staining. Experiments were performed in biological triplicates, analyzing 10,000 cells per experiment. **A**–**C** Pairwise comparison between HR_i_ and/or BPDE-exposed versus non-exposed cells (con) was statistically analyzed using Student’s *t* test (*). Pairwise comparison between HR_i_-pretreated and non-pretreated cells upon exposure to 0.25 BPDE was statistically analyzed using Student’s *t* test (^#^). **D** Phosphorylation of p53, CHK1, CHK2, H2AX, and expression of p21 were assessed via immunodetection; HSP90 or β-Actin were used as internal loading control. **E** mRNA expression of p53-target genes was assessed in VH10tert cells 48 h after BPDE treatment via qPCR; *ACTB* and *GAPDH* were used as internal loading control. Experiments were performed in technical triplicates. Pairwise comparison between HR_i_ and/or BPDE-exposed versus non-exposed cells (con) (*) as well as between BPDE and BPDE/HR_i_ exposed cells (^#^) was statistically analyzed using Student’s *t* test. **A**–**C**/**E** not labeled = not significant, */^#^*p* ≤ 0.05, **/^##^*p* ≤ 0.01, ***/^###^*p* ≤ 0.001, ****/^#####^*p* ≤ 0.0001)

To analyze the relevance of DSBs for the decision between activation of the pro-survival and the pro-cell death pathway, homologous recombination (HR) was inhibited using the specific RAD51 inhibitor RI-1. Upon exposure to 0.25 µM BPDE, HR inhibition clearly enhanced the number of γH2AX foci from 5 to 20 (Fig. 6A), a number already observed upon 2 µM BPDE (Fig. 5F). Furthermore, HR inhibition significantly enhanced cell death activation (Fig. 6B/C). Concerning the activation of the DDR, HR inhibition did not alter Ser15 phosphorylation of p53 but strongly elevated CHK2 and p53^Ser46^ phosphorylation (Fig. 6D). Furthermore, it enhanced γH2AX phosphorylation, decreased CHK1 phosphorylation and abrogated p21 induction. In line with these findings, transcriptional induction of *CDKN1A*, *DDB2, FASR*, *MDM2*, *PUMA* and *XPC* was abrogated and transcription of the pro-apoptotic *NOXA* increased (Fig. 6E).

### Concentration-dependent induction of DNA mutations

**Fig. 7:**
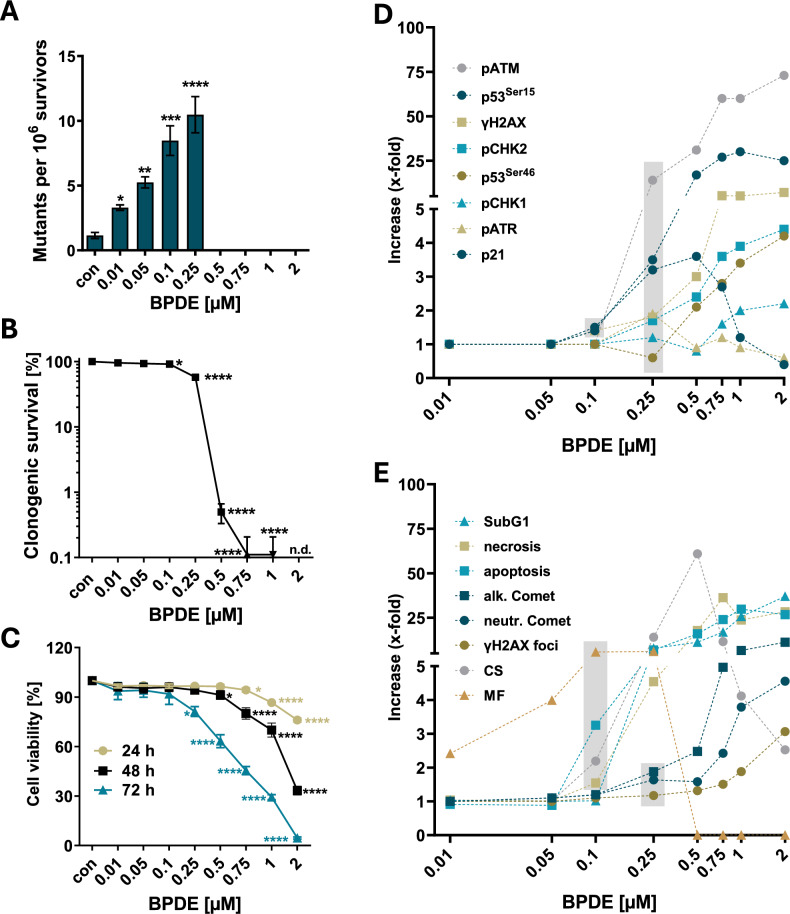
Impact of BPDE on mutation frequency and summary of BPDE-induced PoDs. VH10tert cells were exposed to 0.01–2 µM BPDE. **A** Dose-dependent induction of mutations was measured by HPRT assay. Experiments were performed in biological quadruplicates. **B** Impact on clonogenic survival was measured by colony formation assay. Experiments were performed in biological triplicates, seeding 1000 cells per experiment. n.d. = no colonies detected. **C** Metabolic competence was measured 24 to 72 h after BPDE exposure using MTT assay. Experiments were performed in biological triplicates. **A**–**C** One-way ANOVA with Dunnett correction was performed to compare BPDE-exposed and non-exposed (con) cells. Not labeled = not significant, *p ≤ 0.05, **p ≤ 0.01, ***p ≤ 0.001, ****p ≤ 0.0001). **D** Dose-dependent expression and phosphorylation of DDR factors was analyzed 24 h after exposure via immunodetection and represented in Suppl. Figure 9 and 10. Densitometrical quantification and normalization to the untreated control, which was set to one, is shown. **E** Summary and quantification of cellular responses using the mean values of Fig. 1A/B (SubG1, necrosis, apoptosis), 2A (cellular senescence, CSEN), 5C/D/F (comet assay and H2AX foci) and 7A (mutation frequency, MF). All responses have been normalized to the untreated control, which was set to one. **D**/**E** Artificial lines connecting data points do not represent curve fitting, or modeling procedure. The LOAELs for the different cellular responses are highlighted by a grey box

Among all cellular responses, mutagenicity is most important one, since it triggers tumor formation. Therefore, we analyzed the mutation frequency using the HPRT assay. In brief, the HPRT assay detects gene mutations in VH10tert cells by measuring the loss of HPRT enzyme activity, which allows mutant cells to survive in the presence of 6-thioguanine, while normal cells die. The data revealed a significantly enhanced mutation frequency even upon 0.01 µM BPDE. At concentrations exceeding 0.25 µM, no mutations were observed (Fig. 7A). This went along with the results of the colony formation assay, revealing a complete abrogation of clonogenic survival at these concentrations (Fig. 7B). Using the MTT assay, a strong decrease in cell viability with increasing BPDE concentrations and exposure times was observed only at late time points. (Fig. 7C). Therefore, opposite to the CFA assay, the usage of the MTT assay (performed at early time points after exposure) is not appropriate for selecting concentrations used for mutation analysis since it cannot detect cellular senescence.

Overall, for all cellular endpoints (except for mutations) a PoD was observed with a LOAEL of 0.1–0.25 µM. (Fig. 7D and E for quantification and Suppl. Figs. 9 and 10 for additional immunodetection). Moreover, concentration-dependent differences in the induction of DNA damage, activation of the DDR and cell fate decision were observed at concentrations of 0.25 µM and 2 µM, showing a switch from the protective, senescence-inducing p53^Ser15^ signaling axis to the death-inducing p53^Ser46^ signaling axis of the DDR (Suppl. Fig. 11).

## Discussion

PoDs are central to toxicology because they establish a scientifically grounded threshold at which adverse effects begin to occur. In detail, PoDs mark the dose/concentration where low or no adverse effect is observed, commonly identified as NOAEL, LOAEL, or Benchmark Dose Lower Confidence Limit (BMDL). They serve as a starting point from which safe human exposure limits like Reference Dose (RfD) or Reference Concentration (RfC) are derived. For carcinogenic risks, PoDs, preferably BMDLs, are used to calculate margins of exposure (MoEs) and assess whether current exposures are acceptably low or require risk management actions. Concerning B[a]P-induced non-carcinogenic effects, various NOAEL and LOAEL were observed in human and animal studies for body weight effects, hematological, neurological, hepatic, respiratory, gastrointestinal, reproductive, developmental and renal/bladder effects (Rice et al. 2022). Concerning the carcinogenic activity of B[a]P, based on rat (Kroese et al. 2002) and mouse (Culp et al. 1998) studies, a BMDL_10_ of 0.122 mg B[a]P/kg–bw/day and a MoE of 15,000 was derived (Benford et al. 2010). Moreover, a potency factor for B[a]P of 1.2/mg/kg/day was calculated (Gaylor et al. 2000). In a more recent 2-year oral study in Wistar rats, the lowest dose tested was 3 mg/kg bw/day, which showed increased hepatic foci and considered to be carcinogenic, thus no NOAEL was established below that. However, for tumors, a BMDL_10_ of 1–3 mg/kg/day was derived (Wester et al. 2012). Furthermore, PoDs observed in animal experiments were used to generate organ/system-specific RfDs and overall RfD for benzo[a]pyrene (for review see the “Toxicological Review of Benzo[a]pyrene”, EPA/635/R-17/003Fc, https://iris.epa.gov/static/pdfs/0136_summary.pdf).

In response to genotoxic stress, PoDs for cellular endpoints might be modulated by the DDR, which can either mediate protective or cell-killing outcomes. However, it is not clear whether these different consequences show different PoDs. Here we analyzed whether activation of the DDR and thereby the cellular consequences/endpoints change, depending on the level of DNA damage, and therefore impact PoDs.

### PoDs for BPDE-mediated biological outcomes and mechanism leading from protective to destructive DDR signaling

Here we show that for most BPDE-induced cellular responses, a LOAEL between 0.1 and 0.25 µM was observed (Fig. 7D/E). This holds true for formation of DNA strand breaks, activation of the DDR, induction of cellular senescence and cell death. Importantly, in a very narrow concentration window between 0.5 and 1 µM, the cellular responses completely changed. At low concentrations (0.25 and 0.5 µM), only a weak formation of strand breaks was observed, activating the ATR-CHK1 signaling axis of the DDR and leading to induction of p53^Ser15^ targets involved in NER, cell cycle control and apoptosis, inducing a transient DNA replication block. Moreover, strong induction of senescence was observed in a concentration window of 0.25 to 0.75 µM BPDE. At this concentration, the repair capacity was slightly impaired, leading to formation and persistence of a low number of DNA strand breaks and thereby activation of the ATM-CHK2 axis. At concentrations above 0,75 µM, the repair capacity was completely saturated, leading to increased formation and persistence of DNA strand breaks, massive activation of the ATM-CHK2 axis and cell death. The importance of these DSBs for the switch from protective to destructive DDR was further shown by experiments blocking homologous recombination repair (HR) using a synthetic Rad51 inhibitor. These data clearly show that unrepaired DSBs represent the ultimate trigger for BPDE-induced cell death and that inhibition of HR strongly reduces the threshold level of BPDE. Based on our results, a slight increase in the neutral comet assay, up to the factor of 2 was associated with induction of senescence but was not sufficient to induce cell death. Further, based on γH2AX foci formation, it can be concluded that ~ 3 additional DSBs are sufficient to induce senescence and ~ 15 additional DSBs are the maximum that cells can tolerate before dying.

At toxic concentrations, a switch in the p53 response was observed, leading to repression of p53 targets involved in NER, cell cycle control and apoptosis. The only p53 target still induced was the pro-apoptotic Bcl-2 family member *NOXA*, known to be involved in p53^Ser46^ dependent apoptosis induction (Oda et al. 2000; Schuler et al. 2003). Our data suggest that p53^Ser46^ phosphorylation is not mediated via HIPK2, but rather via ATM and slightly by CHK2. Whether this is a direct or an indirect mechanism, has to be addressed in further studies. However, it has already been shown that the ATM kinase can directly phosphorylate p53 at Ser46 (Enari et al. 2017; Kodama et al. 2010; Liebl and Hofmann 2019). For CHK2, the effect might depend on reduced Ser20 phosphorylation and thereby reduced stability of p53.

Our data concerning concentration-dependent PoDs are in line with a previous study using TK6 cells and BPDE concentrations between 100 and 200 nm BPDE (Piberger et al. 2018). In this case for colony forming ability, a LOAEL of 100 nM BPDE and for transcriptional activation of p53 targets, a LOAEL of 100 to 200 nM BPDE was observed. We should state that in this study ( +)-anti-BPDE was used, whereas we utilized the racemic ( ±)-anti-BPDE which is supposed to show a lover activity, leading to highly comparable results.

### The missing PoD for BPDE-induced mutagenicity

Genotoxic carcinogens are considered to have no PoDs for mutation frequency (Nohmi 2018). In line with this assumption, we observed the formation of mutations already at 0.01 µM BPDE. This is consistent with previous findings from Piberger et al. (Piberger et al. 2018), showing a linear increase in mutagenicity with a frequency of 70 (180)/10^6^ cells at 10 (50) nM using the Pig-A assay and TK6 cells. However, of importance, we could not detect mutations at concentrations above 0.25 µM BPDE. This contrasts with experiments performed in MRC5CV1 cells. In this case, mutations were observed even at higher concentrations, namely 22/10^6^ at 0.2 µM, 25/10^6^ at 0.5 µM and 41/10^6^ at 1 µM BPDE (Hanelt et al. 1997). However, at this concentration, DNA strand breaks were observed and plating efficiency was reduced to less than 10% (Hanelt et al. 1997). Of note, MRC5CV1 cells show no p53 activity due to SV40 transfection and thereby cannot induce a functional p53 response and cellular senescence, which seems to be an important factor in protection against mutations at non-toxic concentrations. In addition, it should be noted that in a previous study we also observed an increased mutation frequency when using 1 µM BPDE (Christmann et al. 2016). However, in this study the overall response to BPDE was much lower with a ~ 4 times lower toxicity and, most importantly, clonogenic survival was still observed at 1 µM. These differences might arise due to different BPDE stocks used in these studies and further indicate that cellular senescence acts as important protective mechanism against formation of mutations at low non-toxic concentrations. Therefore, in case of analyzing mutation frequency, pre-testing systems for cytotoxicity should be selected with care. Thus, metabolic assays are not very reliable since they show only a weak response at senescence-inducing concentrations, especially at early time points (Fig. 7C). Instead, colony formation assays should be used.

The role of DNA adducts in mutagenesis is well documented. Consequently, it has been shown that the stable BPDE-*N*^2^-guanine adduct is responsible for BPDE-induced mutagenesis (Yang et al. 1982) and that removal of these adducts by NER decreases the mutagenic effect of ( ±)-*anti*-BPDE (Yang et al. 1982). Similar to mutation frequency, no PoD was observed regarding formation of DNA adducts (Piberger et al. 2018). Thus, a ~ 100 (~ 500) DNA adducts / 10^8^ cells were observed upon exposure to 10 (50) nm BPDE by HPLF/FD (Piberger et al. 2018). Moreover, also in HCT116 cells, adduct formation was observed using ^32^P-post-labeling in a dose range of 0.1 to 1 µM BPDE (Hockley et al. 2008). In line with this, an increased frequency of DNA adducts and mutations was observed in NER-deficient cells (Lagerqvist et al. 2011).

Overall, our data show that BPDE induces a PoD for most biological endpoints with a LOAEL of 0.1–0.25 µM, and that the switch between protective and destructive outcomes is mediated by unrepaired DNA strand breaks and thereby a switch from p53^Ser15^ to p53^Ser46^ signaling. Whereas p53^Ser46^ signaling clearly depends on DSBs and ATM/CHK2 signaling, the situation is less clear for p53^Ser15^ phosphorylation. We suggest that upon exposure to 0.25 µM BPDE, p53^Ser15^ phosphorylation is caused by replicative stress and mediated by ATR/CHK1 signaling. However, we cannot exclude that the activation of the ATM/CHK2 axis caused by replication fork-associated DSB is involved.

In contrast to DNA repair and DDR activation, for mutation frequency, no PoD was observed. Importantly, we showed that cellular senescence acts as a central protective mechanism against the formation of mutations at low non-toxic concentrations. Of note, for several carcinogens, induction of cellular senescence has been detected in cell system, but also in first animal studies, suggesting that the concentrations necessary for senescence induction can be reached in vivo (Kaina et al. 2026). These senescent cells may negatively impact neighboring cells via activation of the SASP and thereby via constant pro-immune signaling and oxidative stress induced DNA damage (Coppe et al. 2010), which has to be taken into account in future regulatory studies.

## Supplementary Information

Below is the link to the electronic supplementary material.

Supplementary file1 (DOCX 26 KB)

Supplementary file2 (PDF 1859 KB)

## Data Availability

All data supporting the findings of this study are available within the paper.
