# Supplementary material for: Points of Departure for BPDE-induced cellular senescence and cell death are characterized by altered DNA damage signaling and regulated by unrepaired DNA double-strand breaks
Source: Arch Toxicol. 2026 May 21;100(9):4225–44. doi: 10.1007/s00204-026-04426-8 (PMC13454012; doi:10.1007/s00204-026-04426-8)
Supplement: Supplementary file 1 [file 204_2026_4426_MOESM2_ESM.pdf]

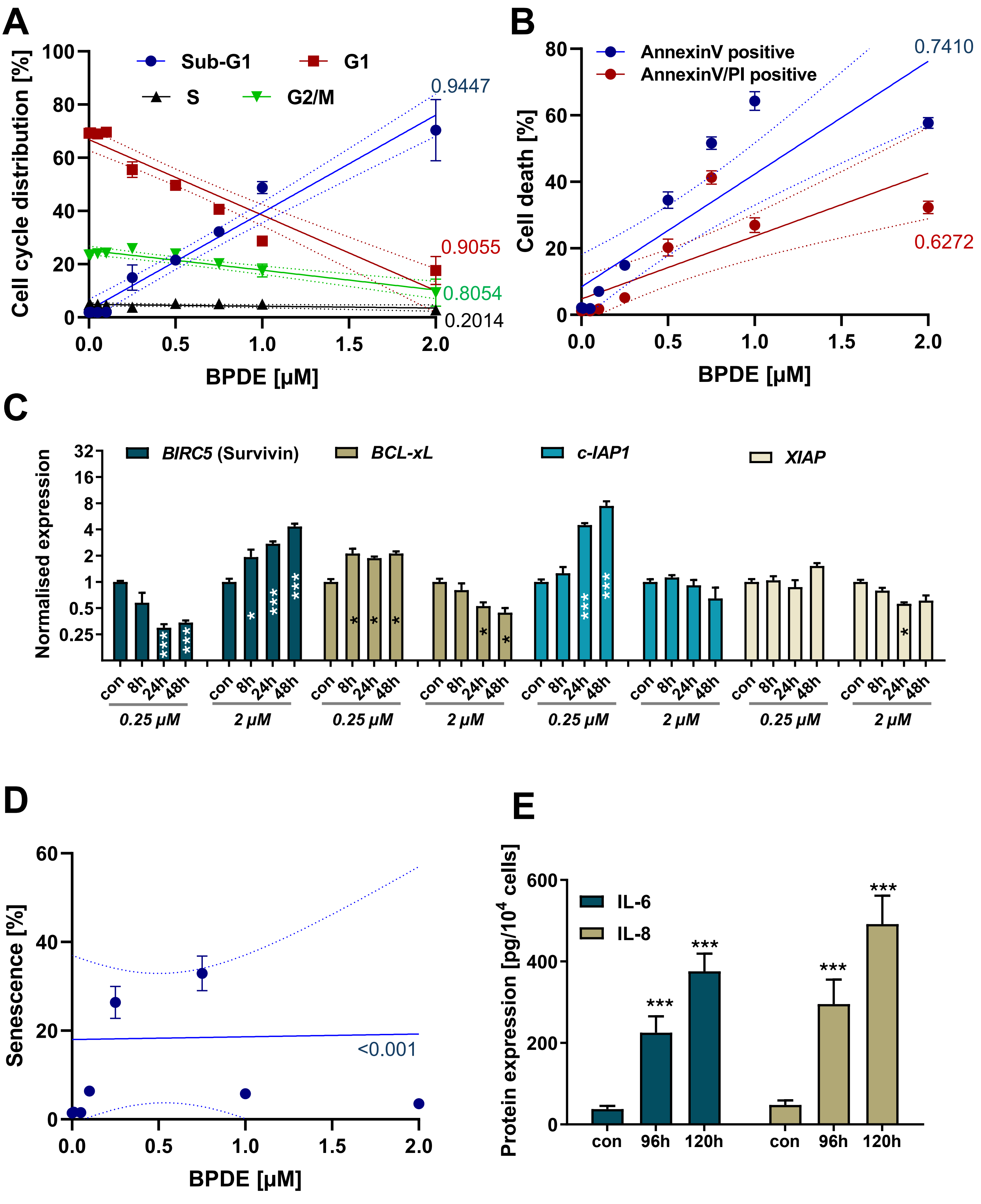
A
Cell cycle distribution [%]
Sub-G1
G1
S
G2/M
0.9447
0.9055
0.8054
0.2014
BPDE [µM]
B
Cell death [%]
AnnexinV positive
AnnexinV/PI positive
0.7410
0.6272
BPDE [µM]
C
Normalised expression
BIRC5 (Survivin)
BCL-xL
c-IAP1
XIAP
con 8h 24h 48h
0.25 µM
2 µM
D
Senescence [%]
<0.001
BPDE [µM]
E
Protein expression [pg/10^4 cells]
IL-6
IL-8
con 96h 120h
***

Suppl Fig. 1

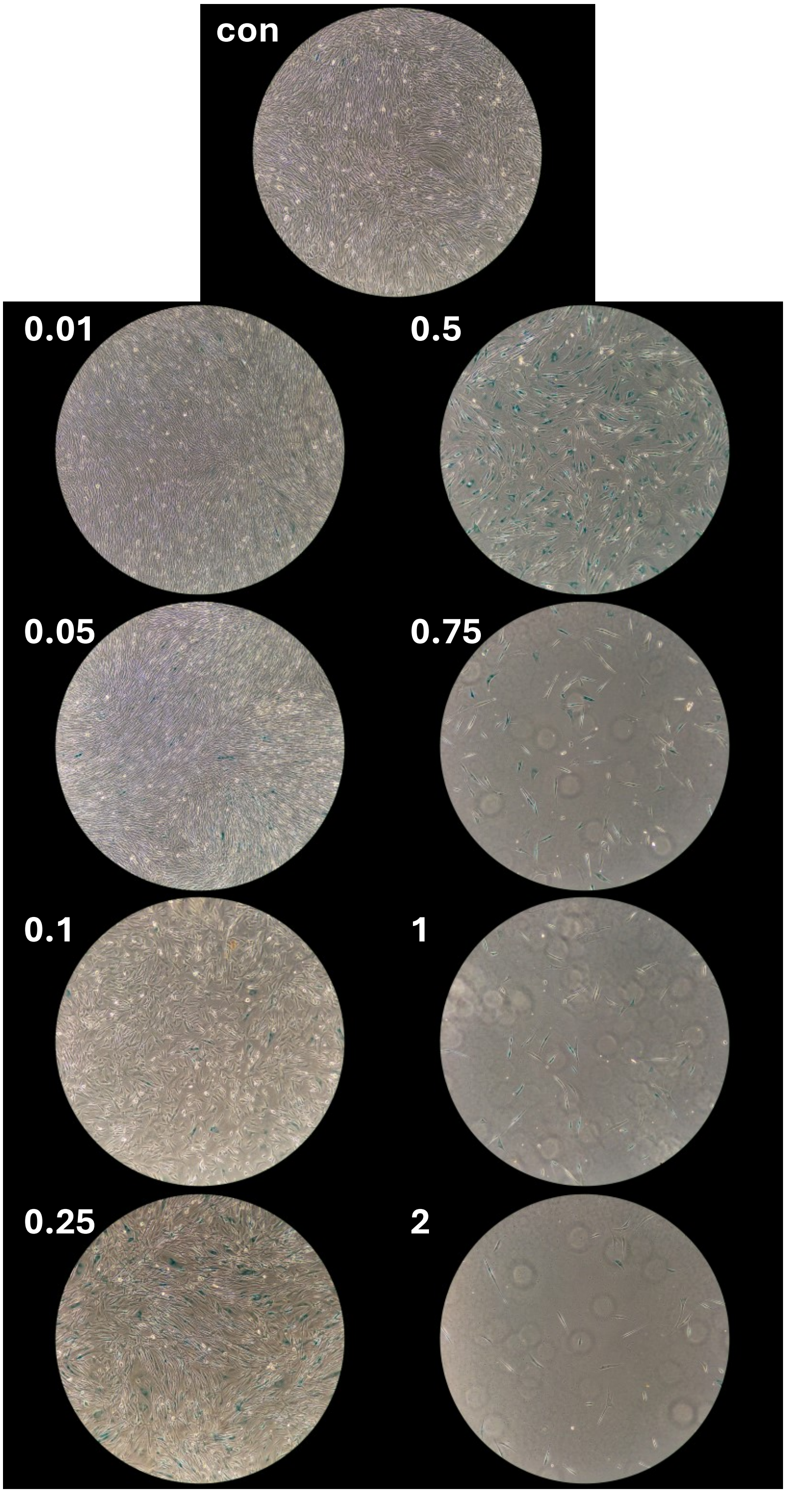

Suppl Fig. 2

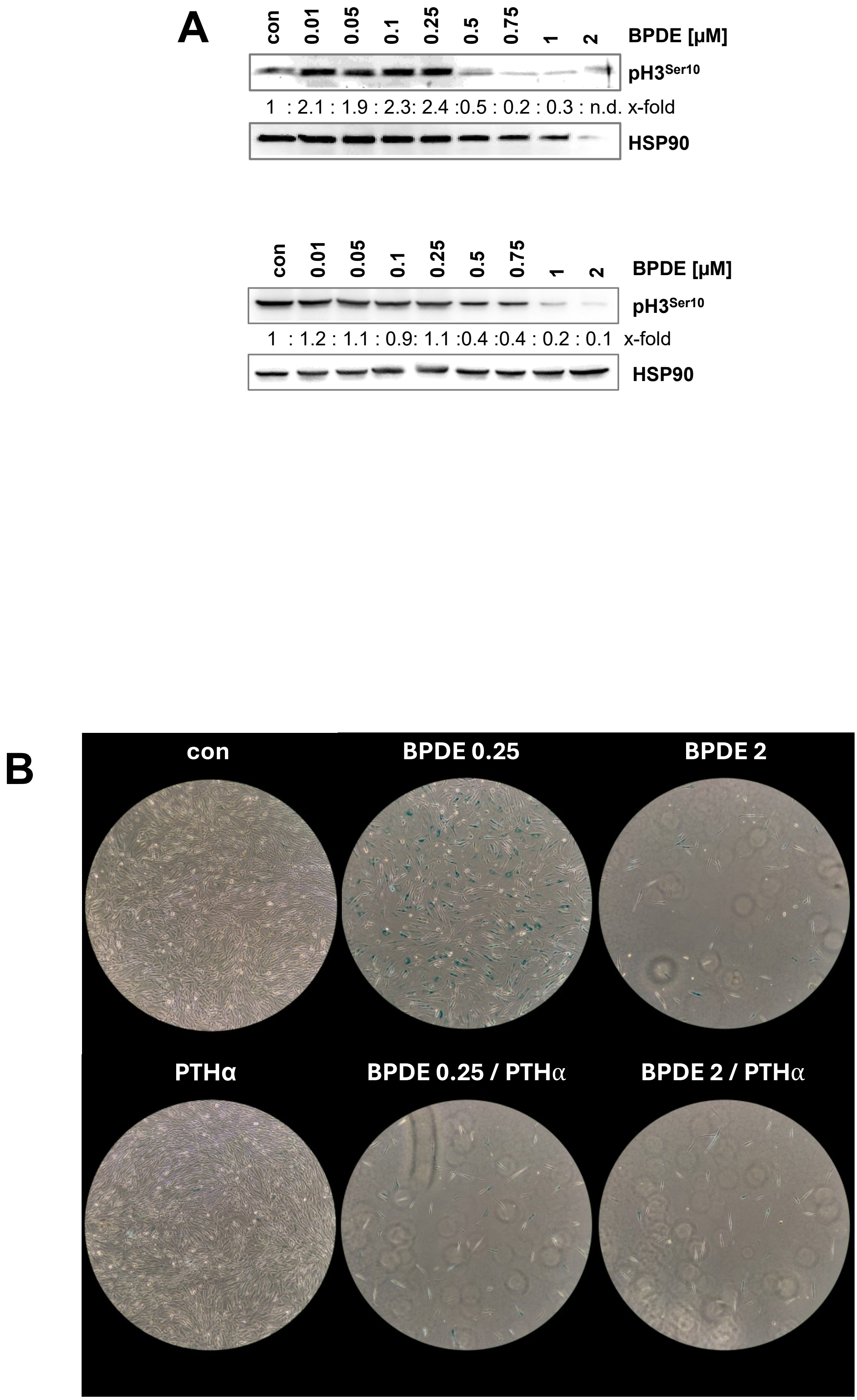
A
con
0.01
0.05
0.1
0.25
0.5
0.75
1
2
BPDE [µM]
pH3Ser10
1 : 2.1 : 1.9 : 2.3: 2.4 :0.5 : 0.2 : 0.3 : n.d. x-fold
HSP90
con
0.01
0.05
0.1
0.25
0.5
0.75
1
2
BPDE [µM]
pH3Ser10
1 : 1.2 : 1.1 : 0.9: 1.1 :0.4 :0.4 : 0.2 : 0.1 x-fold
HSP90
B
con
BPDE 0.25
BPDE 2
PTHα
BPDE 0.25 / PTHα
BPDE 2 / PTHα

Suppl Fig. 3

A

	p53	p53 Ser15	p53 Ser46	p21
con	1	1	1	1
0.25-4	1	9.4	7.6	1.5
0.25-8	1	13.4	5.9	2.9
0.25-16	1.2	16.5	2.1	3.1
con	1	1	1	1
2-4	1	1	6.4	0.1
2-8	0.8	2.6	7.6	0.2
2-16	0,8	4.1	10.1	0.4

	p53	p53 Ser15	p53 Ser46	p21
con	1	1	n.d	1
0.25-16	1.2	1.7	n.d	2.9
0.25-24	1.2	2.2	n.d	3.4
0.25-48	0.9	1.8	n.d	2.7
con	1	1	1	1
2-16	2.6	8.8	2.3	0.3
2-24	2.9	12.1	9.6	0.2
2-48	3.4	9.9	11.2	0.1

B

	pCHK1	pCHK2	pATR	pATM	γH2AX
con	1	1	1	1	1
0.25-4	12.1	4.3	6.4	1	1.3
0.25-8	9.2	3.5	5.4	2.8	1.2
0.25-16	9.3	5.2	4.8	7.5	1
con	1	1	1	1	1
2-4	12.7	6.4	2.3	3.1	18.5
2-8	7.8	7.1	1.5	3.5	20.1
2-16	4.3	2.1	0.0	5.1	19.2

	pCHK1	pCHK2	pATR	pATM	γH2AX
con	1	1	1	1	1
0.25-16	15.3	1	2.3	6.5	0.9
0.25-24	11.2	1.3	2.6	1	1
0.25-48	5.4	1	1.3	1	1.1
con	1	1	1	1	1
2-16	1.1	4.1	1	14.3	14.5
2-24	0.7	3.6	1.3	16.7	19.4
2-48	0.4	2.5	0.4	15.4	20.1

Suppl Fig. 4

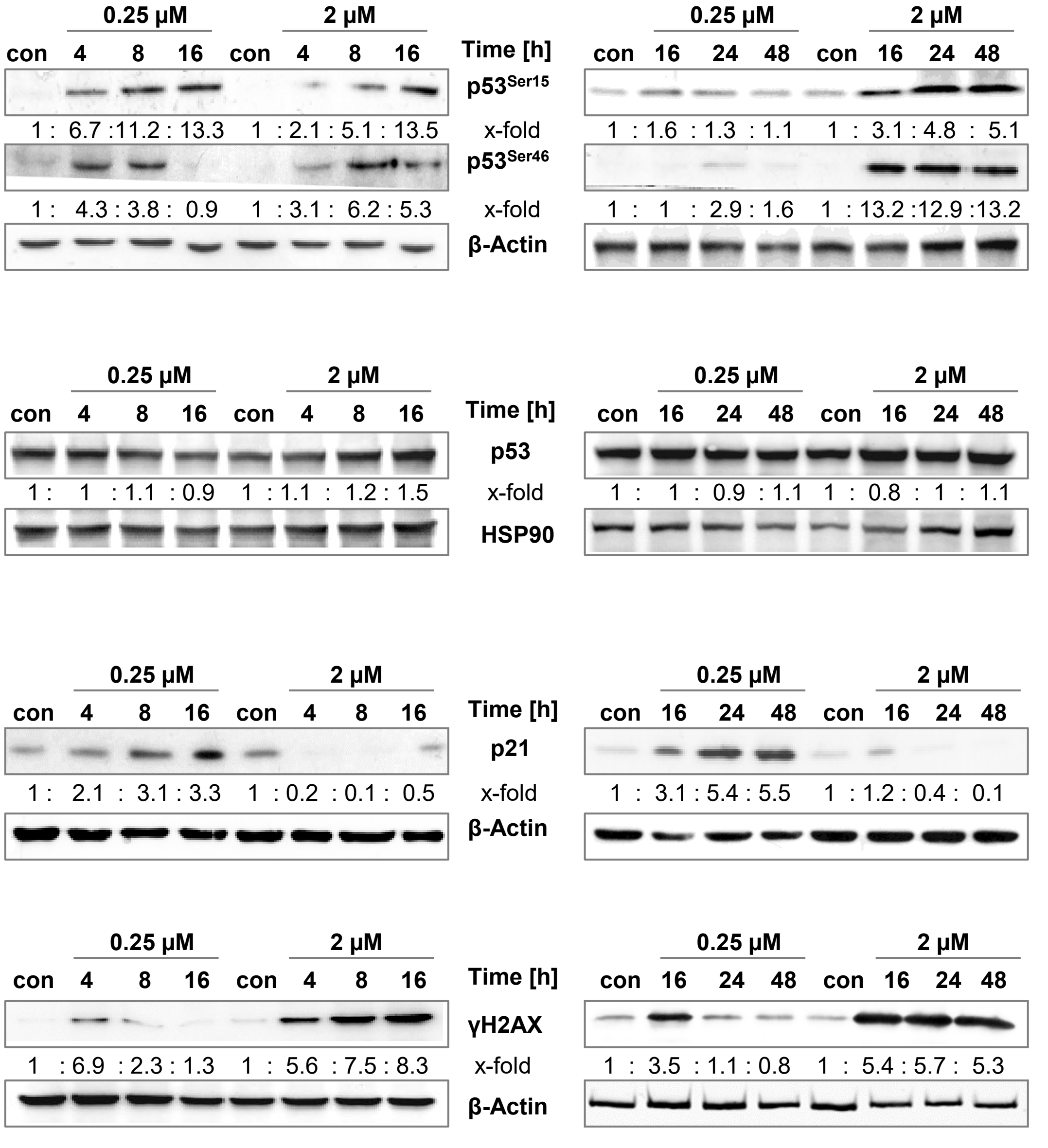
0.25 µM
2 µM
con 4 8 16 con 4 8 16
Time [h]
con 16 24 48 con 16 24 48
p53Ser15
1 : 6.7 :11.2 : 13.3 1 : 2.1 : 5.1 : 13.5
x-fold
1 : 1.6 : 1.3 : 1.1 1 : 3.1 : 4.8 : 5.1
p53Ser46
1 : 4.3 : 3.8 : 0.9 1 : 3.1 : 6.2 : 5.3
x-fold
1 : 1 : 2.9 : 1.6 1 : 13.2 :12.9 :13.2
β-Actin
p53
1 : 1 : 1.1 : 0.9 1 : 1.1 : 1.2 : 1.5
x-fold
1 : 1 : 0.9 : 1.1 1 : 0.8 : 1 : 1.1
HSP90
p21
1 : 2.1 : 3.1 : 3.3 1 : 0.2 : 0.1 : 0.5
x-fold
1 : 3.1 : 5.4 : 5.5 1 : 1.2 : 0.4 : 0.1
β-Actin
γH2AX
1 : 6.9 : 2.3 : 1.3 1 : 5.6 : 7.5 : 8.3
x-fold
1 : 3.5 : 1.1 : 0.8 1 : 5.4 : 5.7 : 5.3
β-Actin

Suppl Fig. 5

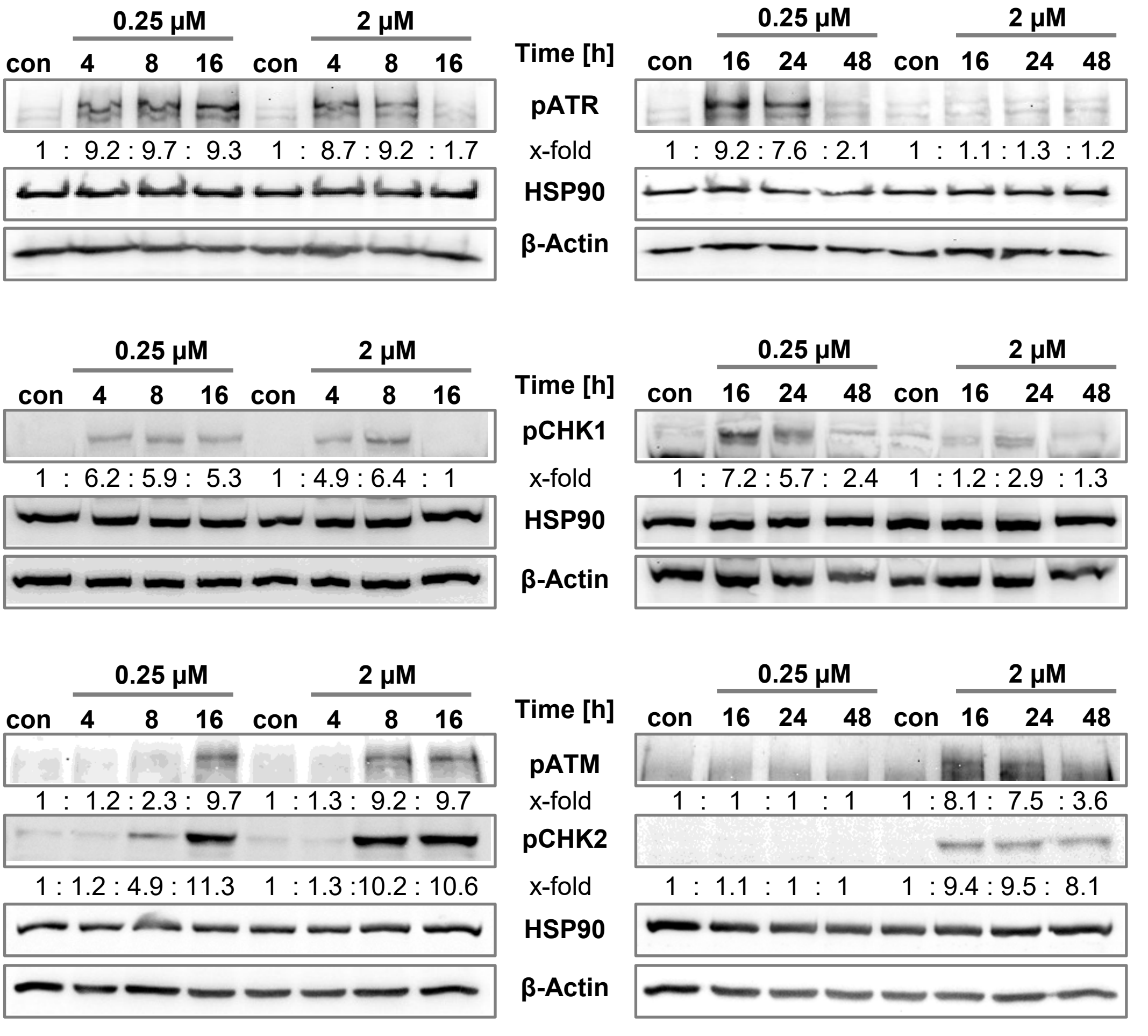
0.25 µM
2 µM
Time [h]
con 4 8 16 con 4 8 16
con 16 24 48 con 16 24 48
pATR
1 : 9.2 : 9.7 : 9.3 1 : 8.7 : 9.2 : 1.7
x-fold
1 : 9.2 : 7.6 : 2.1 1 : 1.1 : 1.3 : 1.2
HSP90
β-Actin
pCHK1
1 : 6.2 : 5.9 : 5.3 1 : 4.9 : 6.4 : 1
1 : 7.2 : 5.7 : 2.4 1 : 1.2 : 2.9 : 1.3
pATM
1 : 1.2 : 2.3 : 9.7 1 : 1.3 : 9.2 : 9.7
1 : 1 : 1 : 1 1 : 8.1 : 7.5 : 3.6
pCHK2
1 : 1.2 : 4.9 : 11.3 1 : 1.3 :10.2 : 10.6
1 : 1.1 : 1 : 1 1 : 9.4 : 9.5 : 8.1

Suppl Fig. 6

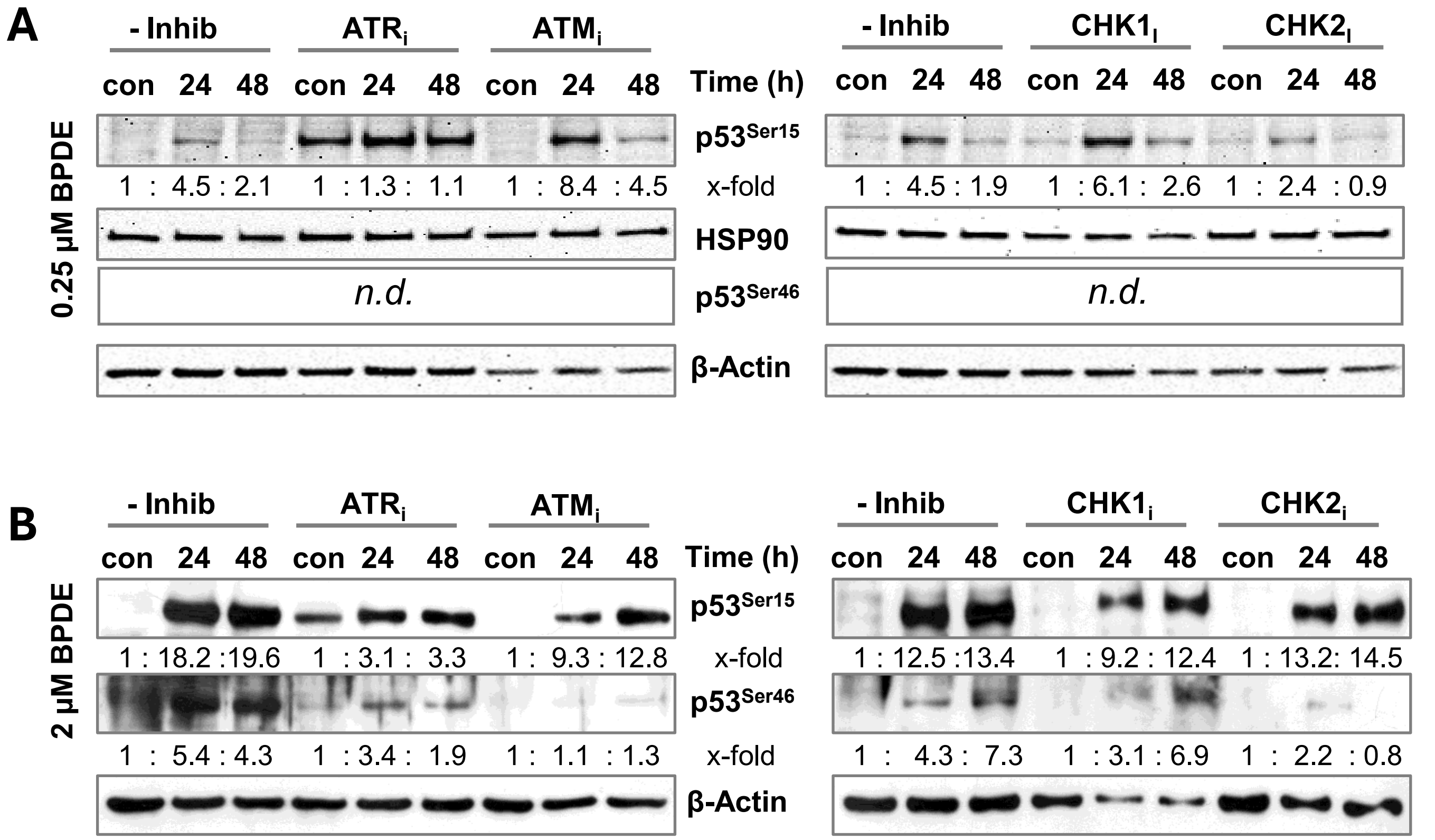
A
B
- Inhib
ATRi
ATMi
CHK1i
CHK2i
con 24 48
Time (h)
p53Ser15
x-fold
HSP90
p53Ser46
β-Actin
n.d.
0.25 µM BPDE
2 µM BPDE
1 : 4.5 : 2.1
1 : 1.3 : 1.1
1 : 8.4 : 4.5
1 : 4.5 : 1.9
1 : 6.1 : 2.6
1 : 2.4 : 0.9
1 : 18.2 :19.6
1 : 3.1 : 3.3
1 : 9.3 : 12.8
1 : 12.5 :13.4
1 : 9.2 : 12.4
1 : 13.2: 14.5
1 : 5.4 : 4.3
1 : 3.4 : 1.9
1 : 1.1 : 1.3
1 : 4.3 : 7.3
1 : 3.1 : 6.9
1 : 2.2 : 0.8

Suppl Fig. 7

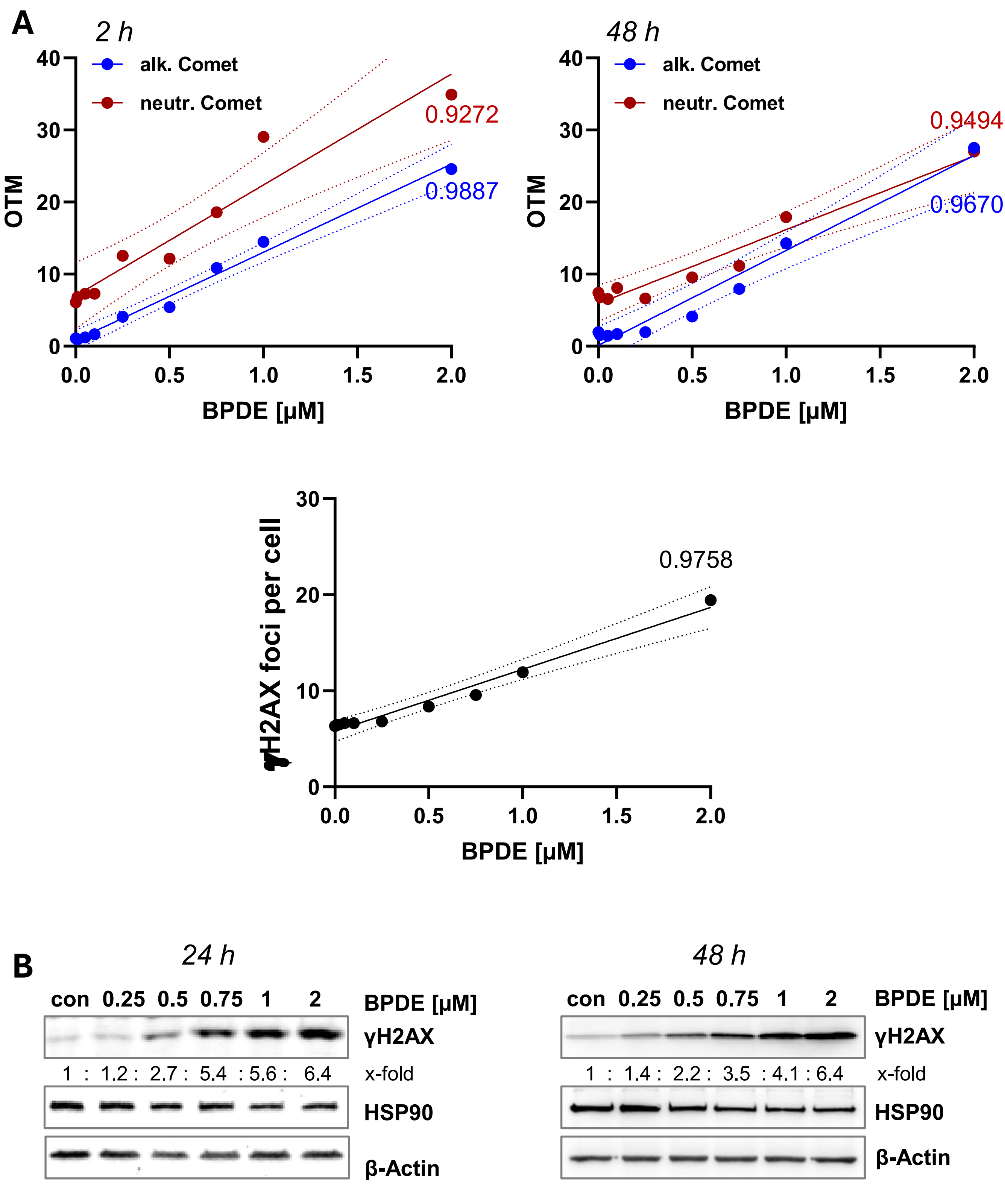

A
2 h
alk. Comet
neutr. Comet
0.9272
0.9887
OTM
BPDE [µM]
48 h
alk. Comet
neutr. Comet
0.9494
0.9670
OTM
BPDE [µM]
0.9758
γH2AX foci per cell
BPDE [µM]
B
24 h
con 0.25 0.5 0.75 1 2
BPDE [µM]
γH2AX
1 : 1.2 : 2.7 : 5.4 : 5.6 : 6.4
x-fold
HSP90
β-Actin
48 h
con 0.25 0.5 0.75 1 2
BPDE [µM]
γH2AX
1 : 1.4 : 2.2 : 3.5 : 4.1 : 6.4
x-fold
HSP90
β-Actin

Suppl Fig. 8

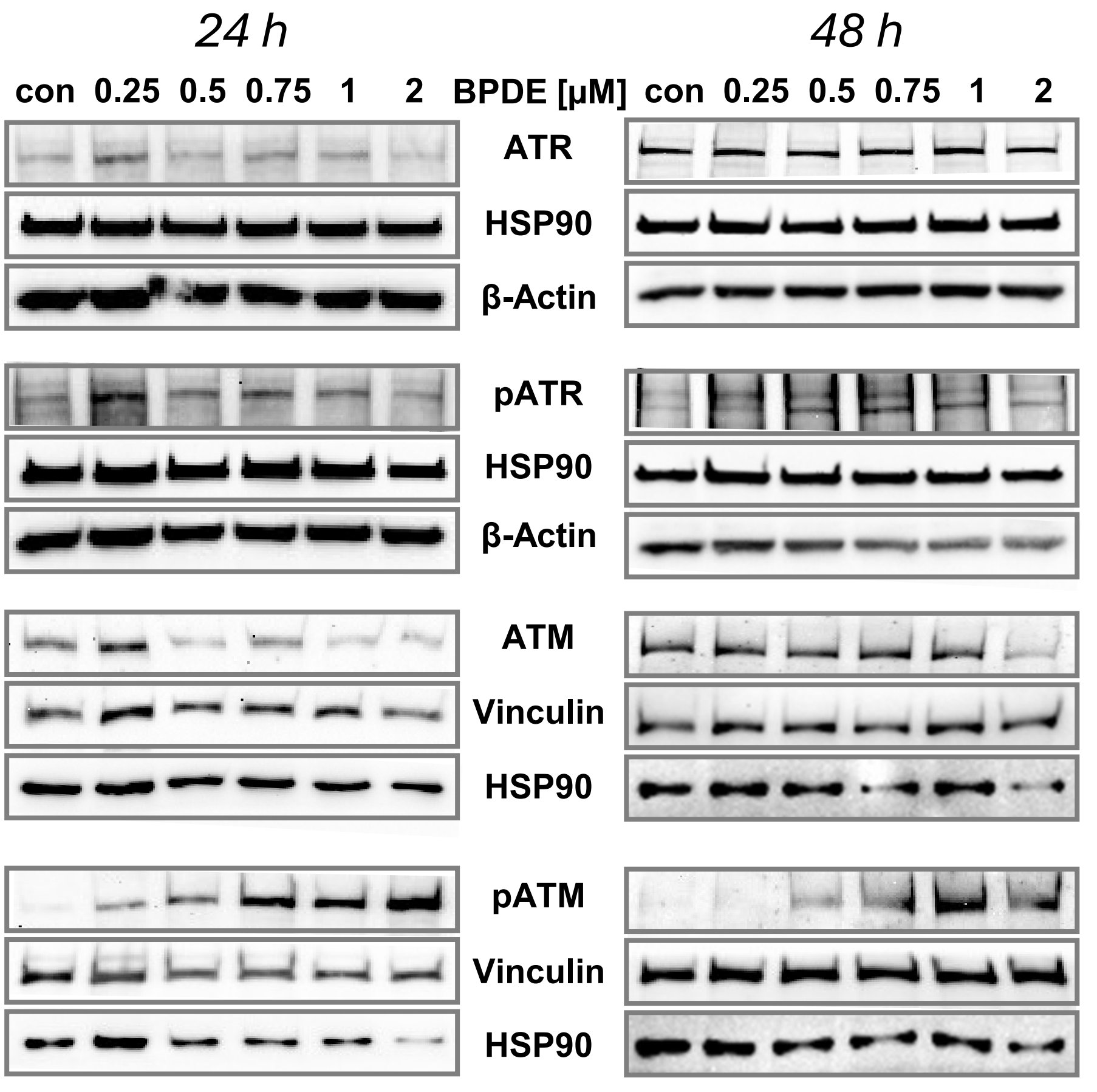

24 h
48 h
con 0.25 0.5 0.75 1 2 BPDE [µM] con 0.25 0.5 0.75 1 2
ATR
HSP90
β-Actin
pATR
HSP90
β-Actin
ATM
Vinculin
HSP90
pATM
Vinculin
HSP90

Suppl Fig. 9 A

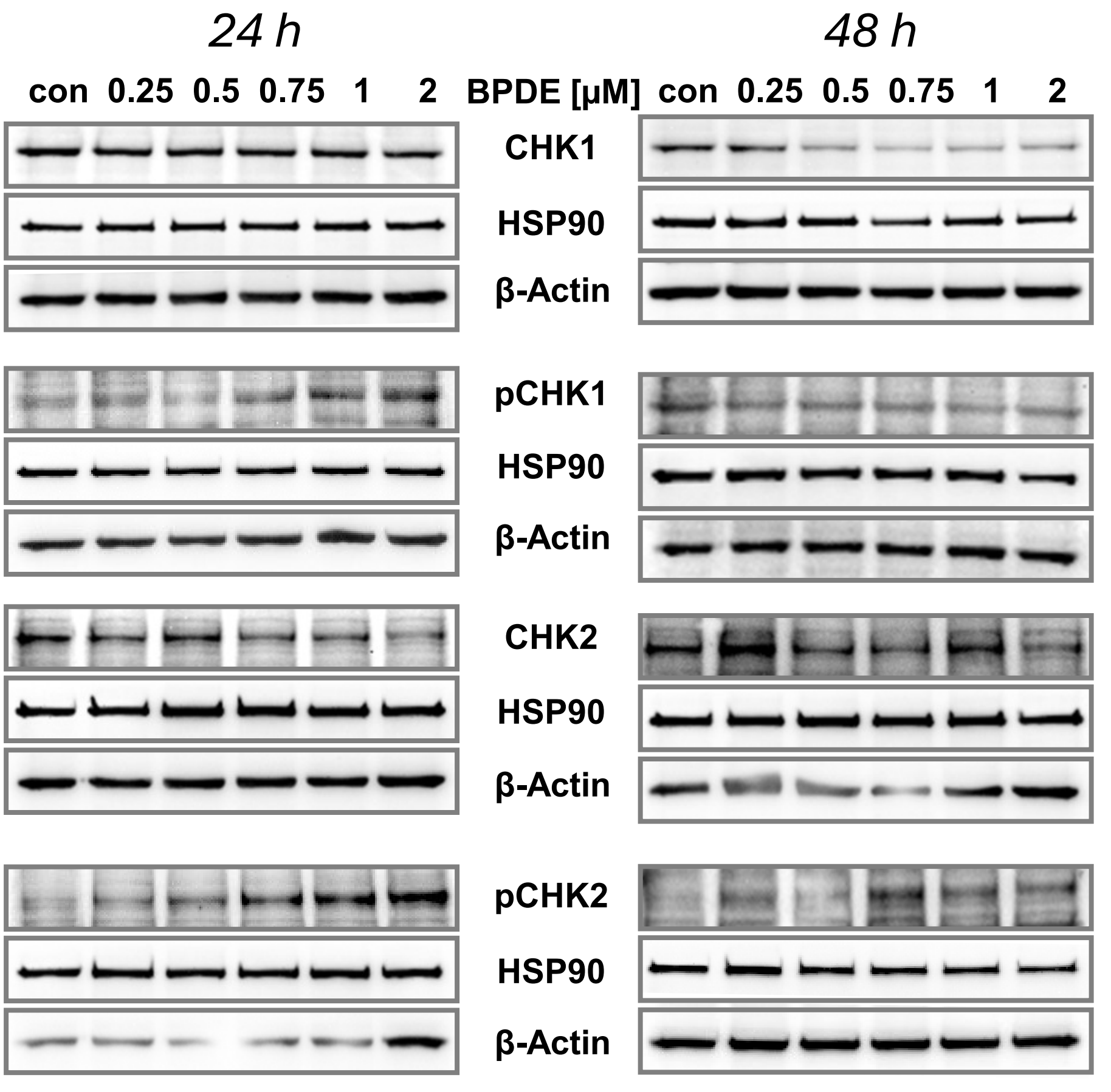
24 h
48 h
con 0.25 0.5 0.75 1 2
BPDE [µM]
con 0.25 0.5 0.75 1 2
CHK1
HSP90
β-Actin
pCHK1
HSP90
β-Actin
CHK2
HSP90
β-Actin
pCHK2
HSP90
β-Actin

Suppl Fig. 9B

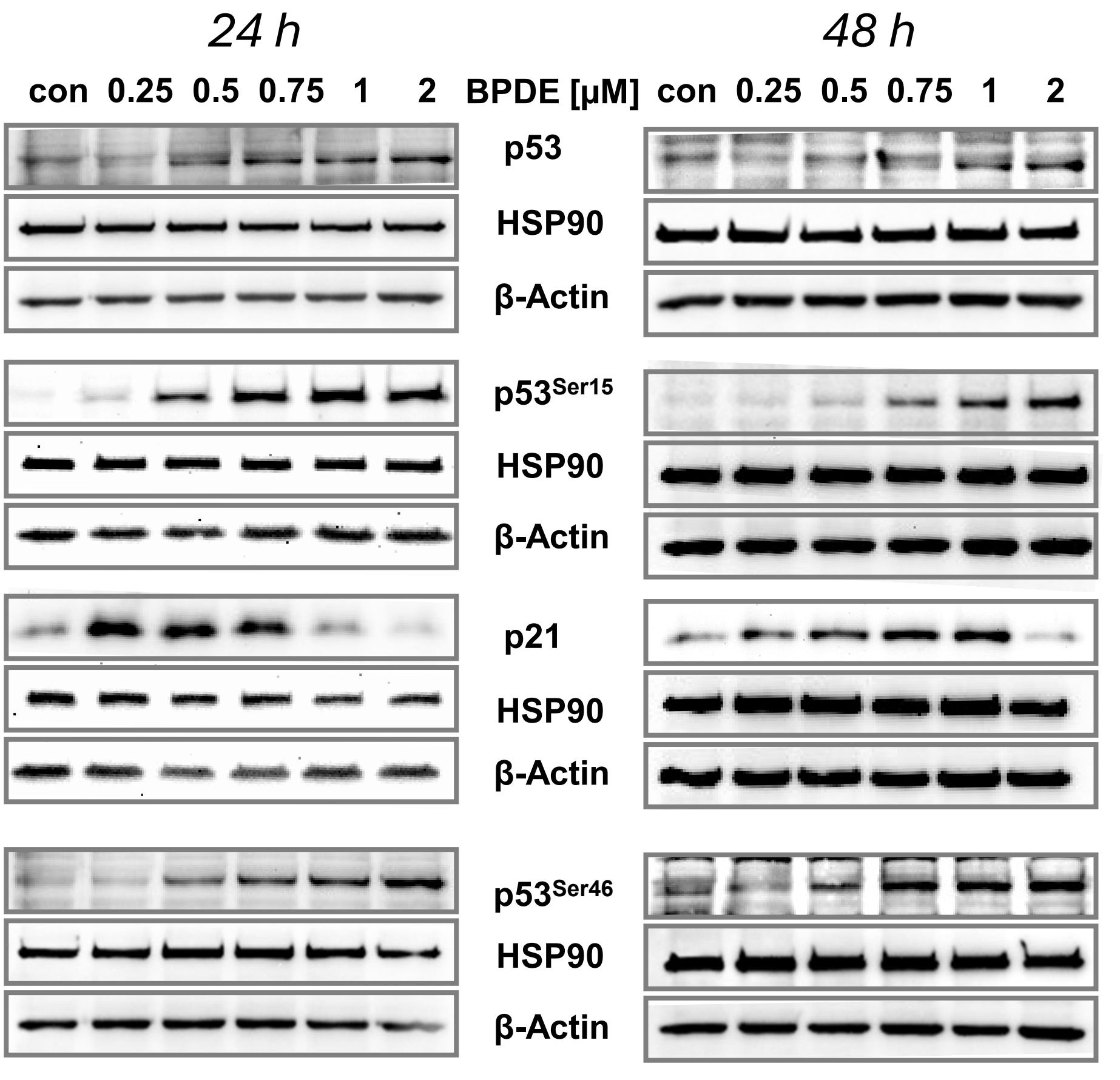
24 h
48 h
con 0.25 0.5 0.75 1 2 BPDE [µM] con 0.25 0.5 0.75 1 2
p53
HSP90
β-Actin
p53Ser15
HSP90
β-Actin
p21
HSP90
β-Actin
p53Ser46
HSP90
β-Actin

Suppl Fig. 9C

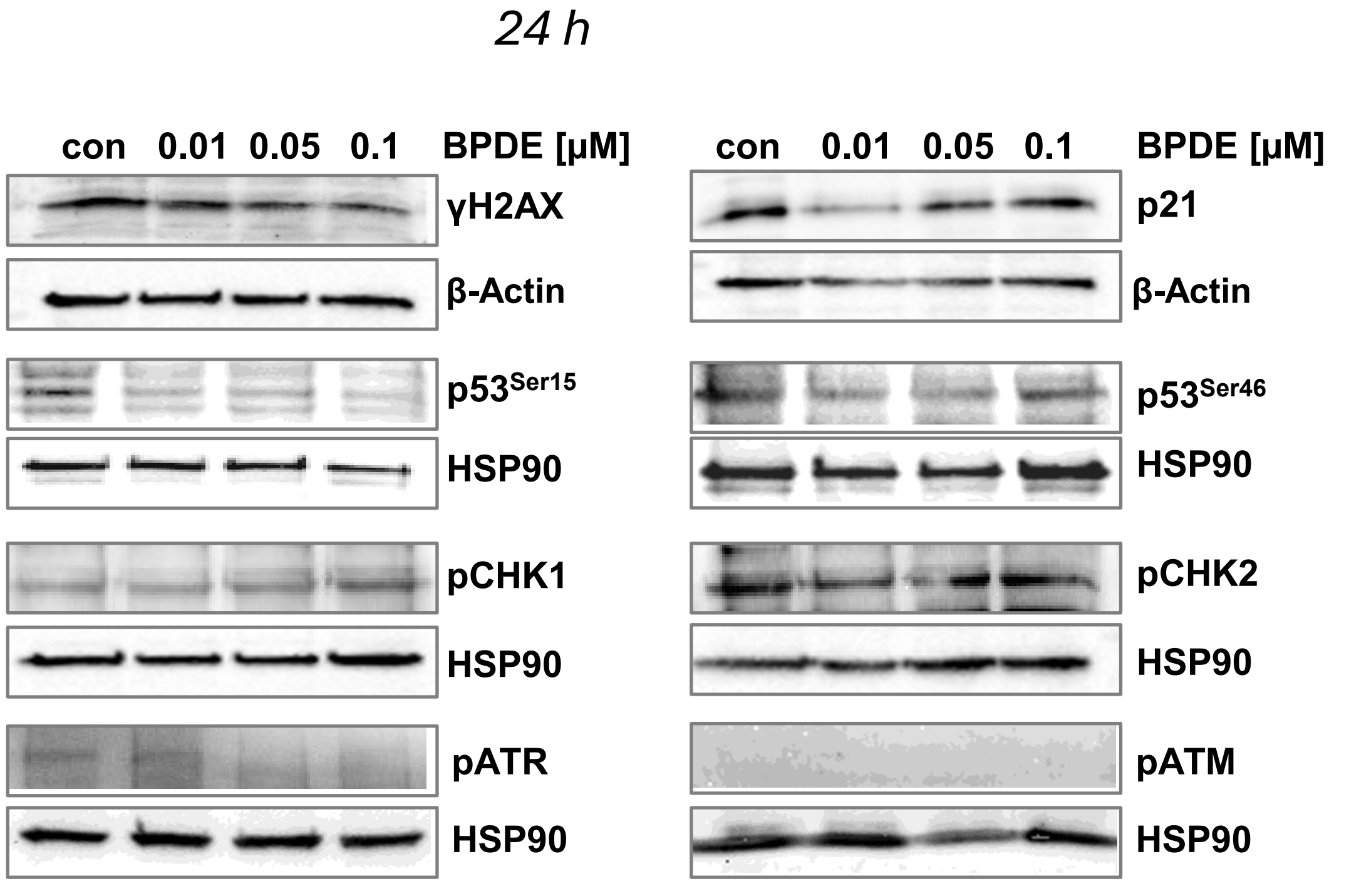
24 h
con 0.01 0.05 0.1 BPDE [µM]
γH2AX
β-Actin
p53Ser15
HSP90
pCHK1
HSP90
pATR
HSP90
con 0.01 0.05 0.1 BPDE [µM]
p21
β-Actin
p53Ser46
HSP90
pCHK2
HSP90
pATM
HSP90

Suppl Fig. 10

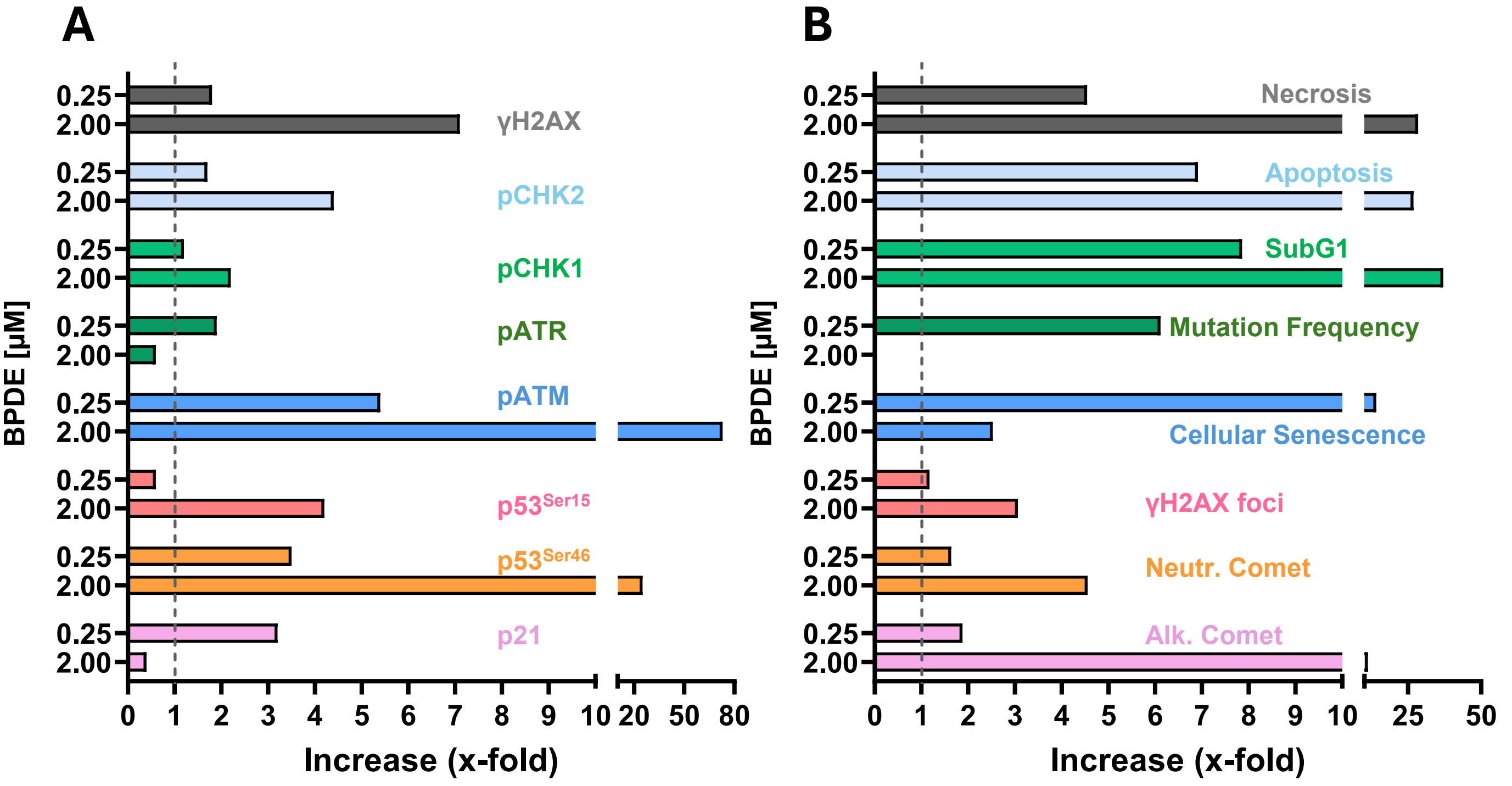
A
B
BPDE [µM]
0.25
2.00
γH2AX
pCHK2
pCHK1
pATR
pATM
p53Ser15
p53Ser46
p21
Increase (x-fold)
0 1 2 3 4 5 6 7 8 9 10 20 50 80
Necrosis
Apoptosis
SubG1
Mutation Frequency
Cellular Senescence
γH2AX foci
Neutr. Comet
Alk. Comet
0 1 2 3 4 5 6 7 8 9 10 25 50

Suppl Fig. 11
