# Supplementary material for: Points of Departure for BPDE-induced cellular senescence and cell death are characterized by altered DNA damage signaling and regulated by unrepaired DNA double-strand breaks
Source: Arch Toxicol. 2026 May 21;100(9):4225–44. doi: 10.1007/s00204-026-04426-8 (PMC13454012; doi:10.1007/s00204-026-04426-8)
Supplement: Supplementary file 2 [file 204_2026_4426_MOESM1_ESM.docx]

**Suppl. Table 1: Primers used within the study**

| qPCR Primer | Sequence (5‘-3‘) |
| --- | --- |
| ACTB-for | tggcatccacgaaactacc |
| ACTB-rev | gtgttggcgtacaggtctt |
| BAX-for | ATCAGATGTGGTCTATAATG |
| BAX-rev | CAGAAGGCACTAATCAAG |
| BCL_XL_-for | AAGCGTAGACAAGGAGAT |
| BCL_XL_-rev | TAGGTGGTCATTCAGGTAA |
| BIRC5-for | ATGACTTGTGTGTGATGA |
| BIRC5-rev | GTTTGTGCTATTCTGTGAA |
| CDKN1a (p21)-for | ACTCTCAGGGTCGAAAAC |
| CDKN1a (p21)-rev | CGGCGTTTGGAGTGGTA |
| c-IAP1-for | TCACTCCCAGACTCTTTCCA |
| c-IAP1-rev | CCCCGTGTTCTACAAGTGTC |
| DDB2-for | TGTAGCCTGGATGTGTCT |
| DDB2-rev | GCATTCTGAGATTCCAAAGC |
| E2F1-for | ATGGTGATCAAAGCCCCTCC |
| E2F1-rev | AAACATCGATCGGGCCTTGT |
| FASR-for | AGAACTTGGAAGGCCTGCAT |
| FASR-rev | CTGGTTCATCCCCATTGACT |
| FOXM1-for | GCAGCATCAAGCAAGAGATG |
| FOXM1-rev | GCCGCTCAGACACAGAGTTC |
| GADD45a-for | ACGATCACTGTCGGGGTGTA |
| GADD45a-rev | CACGTTATCGGGGTCGACGTT |
| GAPDH-for | catgagaagtatgacaacag |
| GAPDH-rev | atgagtccttccacgata |
| MDM2-for | AGGCTATAATCTTCTGAGTC |
| MDM2-rev | ATCTTGATGCTGGTGTAA |
| MYBL2-for | TGCTGTGGGTTCTAGCCTCT |
| MYBL2-rev | AGATGGTTCCTCAGGGAGGT |
| NOXA-for | GAGATGCCTGGGAAGAAGG |
| NOXA-rev | GCATCCCAATCGCAAATC |
| PUMA-for | GACGACCTCAACGCACAGTA |
| PUMA-rev | CTGGGTAAGGGCAGGAGTC |
| XIAP-for | CTGAGCCAGATCAAAGTATG |
| XIAP-rev | CCGAAGAGAAACCACATTT |
| XPC-for | ACACCTACTACCTCTCAA |
| XPC-rev | TAAATAGCAAAATCTCCTTTCC |
| XPV-for | ACATTAATCTCCATCACTTCA |
| XPV-rev | ATCTTCTACTGGCACAAG |

**Suppl. Table 2: Antibodies used within the study**

| Protein | Molecular  weight (kDa) | Antibody | RRID | Company |
| --- | --- | --- | --- | --- |
| β-Actin | 42 | sc-47778 | AB_2714189 | Santa Cruz Biotechnology |
| Caspase6 | 35 18 cleaved | 9762 | AB_10829240 | Cell Signaling Technology |
| HIPK2 rb1 | 130 | selfmade |  | Winter, M., Sombroek, D., Dauth, I., Moehlenbrink, J., Scheuermann, K., Crone, J. & Hofmann, T. G. Control of HIPK2 stability by ubiquitin ligase Siah-1 and checkpoint kinases ATM and ATR. *Nat Cell Biol* **10,** 812–824; 10.1038/ncb1743 (2008). |
| HSP90 | 90 | sc-13119 | AB_675659 | Santa Cruz Biotechnology |
| γH2AX^Ser139^ | 15 | 9718 | AB_2118009 | Cell Signaling Technology |
| p21 | 21 | ab109520 | AB_10860537 | Abcam |
| p53 | 53 | sc-100 | AB_628087 | Santa Cruz Biotechnology |
| p53^Ser15^ | 53 | 9286 | AB_331741 | Cell Signaling Technology |
| p53^Ser46^ | 53 | 558245 | AB_647288 | BD Biosciences |
| pH3^Ser10^ | 15 | 9701 | AB_331535 | Cell Signaling Technology |
| pATM (Ser1981) | 350 | ab81292 | AB_1640207 | Abcam |
| pATR (Thr1989) | 300 | 30362 | AB_2798992 | Cell Signaling Technology |
| pCHK1 (Ser345) | 56 | 2348 | AB_331212 | Cell Signalling Technology |
| pCHK2 (Thr68) | 62 | 2661 | AB_331479 | Cell Signalling Technology |
| pHIPK2 | 130 | selfmade |  | Bitomsky, N., Conrad, E., Moritz, C., Polonio-Vallon, T., Sombroek, D., Schultheiss, K., Glas, C., Greiner, V., Herbel, C., Mantovani, F., del Sal, G., Peri, F. & Hofmann, T. G. Autophosphorylation and Pin1 binding coordinate DNA damage-induced HIPK2 activation and cell death. *Proceedings of the National Academy of Sciences of the United States of America* **110,** E4203-12; 10.1073/pnas.1310001110 (2013). |
| SIAH1 | 34 | PA5-29682 | AB_2547157 | Thermo Fisher Scientific |
| Vincullin | 117 | sc-73614 | AB_2941767 | Santa Cruz Biotechnology |
| 53BP1 | 213 | ab21083 | AB_722496 | Abcam |
|  |  |  |  |  |

| Protein | Antibody | RRID | Company |
| --- | --- | --- | --- |
| HRP conjugated anti-mouse | KCB002 | AB_10703407 | Rockland |
| HRP conjugated anti-rabbit | KCB003 | AB_10702763 | Rockland |
| Alexa Fluor 488 goat-anti-mouse | A11017 | AB_143160 | Thermo Fisher Scientific Invitrogen |
| Cy3 goat-anti-rabbit | ab97075 | AB_10679955 | Abcam |

**Legends to Figures**

**Suppl Fig. 1. BPDE-induced cell death and regulation of anti-apoptotic factors and IL-6/IL-8.** **A)** Cell cycle distribution and cell death induction were assessed 96 h after BPDE treatment of VH10tert cells *via* flow cytometry using PI staining. Linear regression and R square of data from Fig. 1A are shown. **B)** Dose-dependent induction of apoptosis and necrosis in VH10tert cells was analyzed *via* flow cytometry using Annexin V/PI staining 96 h after BPDE treatment. Linear regression and R square of data from Fig.1B are shown. **C)** VH10tert cells were exposed to 0.25 or 2 µM BPDE for 8-48 h. mRNA expression of anti-apoptotic factors was assessed via qPCR; ACTB and GAPDH were used as internal loading control. Experiments were performed in technical triplicates. Pairwise comparison between BPDE-exposed and non-exposed cells, was statistically analyzed using Student's *t* test. **D)** Senescence was assessed 120 h after BPDE treatment of VH10tert cells *via* β-Gal staining. Linear regression and R square of data from Fig. 2A are shown. **E)** VH10tert cells were exposed to 0.25 µM BPDE. The amount of the SASP factors IL-6 and IL-8 was quantified after 96 and 120 h by the human IL-6 and IL-8 ELISA Kit from ImmunoTools (Friesoythe, Germany) according to the manufacturer protocol. Experiment was performed in technical triplicates. **C/E)** Pairwise comparison between BPDE-exposed and non-exposed cells was statistically analyzed using Student's *t* test. Not labeled = not significant, *p ≤ 0.05, **p ≤ 0.01, ***p ≤ 0.001, ****p ≤ 0.0001).

**Suppl Fig. 2. Dose-dependent induction of senescence upon BPDE.** Senescence was assessed 120 h after dose-dependent BPDE treatment of VH10tert cells *via* β-Gal staining. Representative images related to Fig. 2A are shown.

**Suppl Fig. 3. Dose-dependent phosphorylation of Histone 3 and induction of senescence upon BPDE.** **A)** Expression of pH3^Ser10^ protein was measured 120 h after BPDE treatment *via* immunodetection; HSP90 was used as internal loading control. Expression was densitometrical analyzed and the control was set to one. n.d.= not determined **B)** VH10tert cells were treated with PTHα 1 h prior to BPDE exposure and cellular senescence was assessed 120 h after BPDE treatment of VH10tert cells *via* β-Gal staining. Representative images related to Fig. 2D are shown.

**Suppl Fig. 4. Activation of the DDR.** Expression and phosphorylation of p53, CHK1, CHK2, ATR, ATM and H2AX as well as expression of p21 was measured at different time points after treatment with 0.25 and 2 µM BPDE *via* immunodetection. Densitometrical quantification of Fig. 2F and G is shown; the control was set to one.

**Suppl Fig. 5. Activation of the DDR.** Expression and phosphorylation of p53 and H2AX as well as expression of p21 was measured at different time points after treatment with 0.25 and 2 µM BPDE *via* immunodetection. HSP90 and β-Actin were used as internal loading control. Densitometrical quantification is shown; the control was set to one.

**Suppl Fig. 6. Activation of the DDR.** Expression and phosphorylation of CHK1, CHK2, ATR and ATM, was measured at different time points after treatment with 0.25 and 2 µM BPDE *via* immunodetection. HSP90 and β-Actin were used as internal loading control. Densitometrical quantification is shown; the control was set to one.

**Suppl Fig. 7. Impact of the DDR on BPDE-induced p53 phosphorylation.** VH10tert cells was treated with inhibitors against ATR, ATM, CHK1 and CHK2 1 h prior to exposure to 0.25 **(A)** or 2 µM **(B)** BPDE. Phosphorylation of p53 at serine 15 and serine 46 was assessed *via* immunodetection and HSP90 or β-Actin were used as internal loading control. Densitometrical quantification is shown; the control was set to one.

**Suppl Fig. 8. BPDE-induced formation of strand breaks.** **A)** Induction of strand breaks were assessed 2 and 48 h after BPDE treatment using the alkaline and neutral comet assay, as well as γH2AX foci formation. Linear regression and R square of data from Fig. 5A-D/F are shown. **B)** VH10tert cells were treated with different concentrations of BPDE for 24 or 48 h. Expression and phosphorylation of H2AX was assessed *via* immunodetection. HSP90 and β-Actin were used as internal loading control. Densitometrical quantification is shown; the control was set to one.

**Suppl Fig. 9. Activation of the DDR.** VH10tert cells were treated with different concentrations of BPDE for 24 or 48 h. A) Expression and phosphorylation of ATR and ATM was assessed *via* immunodetection and HSP90, Vinculin or β-Actin were used as internal loading control. B) Expression and phosphorylation of CHK1 and CHK2 was assessed *via* immunodetection and HSP90 or β-Actin were used as internal loading control. C) Expression and phosphorylation of p53 and expression of p21 were assessed *via* immunodetection and HSP90 or β-Actin were used as internal loading control.

**Suppl Fig. 10. Activation of the DDR at low concentrations.** VH10tert cells were treated with different concentrations of BPDE for 24 h. Expression and phosphorylation of ATR, ATM, CHK1, CHK2, H2AX, p53 and expression of p21 was assessed *via* immunodetection. HSP90 or β-Actin were used as internal loading control.

**Suppl Fig. 11. Comparison of the cellular outcome at 0.25 and 2 µM BPDE. A)** Dose-dependent expression and phosphorylation of DDR factors was analyzed 24 h after exposure *via* immunodetection. Densitometrical quantification of experiments presented in Suppl. Fig. 9 and 10 (0.25 and 2 µM) and normalization to the untreated control are shown. **B)** Summary and quantification of cellular responses using the mean values for 0.25 and 2 µM of Fig. 1A/B (SubG1, necrosis, apoptosis), 2A (cellular senescence, CS), 5C/D/F (comet assay and H2AX foci) and 7A (mutation frequency, MF). All responses have been normalized to the untreated control, which was set to one.
